# Synthetic Biology Toolbox, Including a Single-Plasmid CRISPR-Cas9 System to Biologically Engineer the Electrogenic, Metal-Resistant Bacterium *Cupriavidus metallidurans* CH34

**DOI:** 10.1021/acssynbio.2c00130

**Published:** 2022-10-24

**Authors:** Federico Turco, Marco Garavaglia, Rob Van Houdt, Phil Hill, Frankie J. Rawson, Katalin Kovacs

**Affiliations:** † School of Pharmacy, Biodiscovery Institute, University of Nottingham, Nottingham NG7 2RD, United Kingdom; ‡ BBSRC/EPSRC Synthetic Biology Research Centre, School of Life Sciences, Biodiscovery Institute, University of Nottingham, Nottingham NG7 2RD, United Kingdom; § Microbiology Unit, Belgian Nuclear Research Centre (SCK CEN), Boeretang 200, 2400 Mol, Belgium; ∥ School of Biosciences, The University of Nottingham, Sutton Bonington Campus, Leicestershire LE12 5RD, United Kingdom; ⊥ Bioelectronics Laboratory, School of Pharmacy, University of Nottingham, Nottingham NG7 2RD, United Kingdom; # Division of Molecular Therapeutics and Formulations, School of Pharmacy, University of Nottingham, Nottingham NG7 2RD, United Kingdom

**Keywords:** C. metallidurans CH34, CRISPR, Cas9, direct electron transfer, extracellular electron transfer, genome editing, promoter libraries, riboswitch, mediated electron transfer, microbial fuel cells, type IV pili

## Abstract

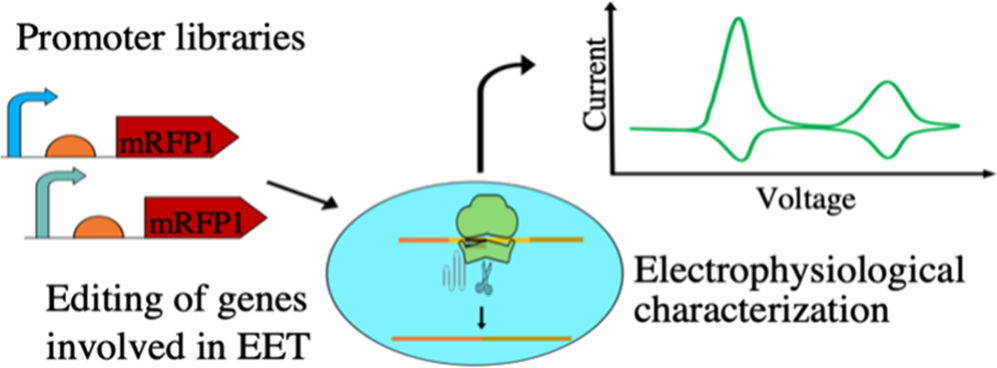

*Cupriavidus metallidurans* CH34 exhibits extraordinary metabolic versatility, including chemolithoautotrophic growth; degradation of BTEX (benzene, toluene, ethylbenzene, xylene); high resistance to numerous metals; biomineralization of gold, platinum, silver, and uranium; and accumulation of polyhydroxybutyrate (PHB). These qualities make it a valuable host for biotechnological applications such as bioremediation, bioprocessing, and the generation of bioelectricity in microbial fuel cells (MFCs). However, the lack of genetic tools for strain development and studying its fundamental physiology represents a bottleneck to boosting its commercial applications. In this study, inducible and constitutive promoter libraries were built and characterized, providing the first comprehensive list of biological parts that can be used to regulate protein expression and optimize the CRISPR-Cas9 genome editing tools for this host. A single-plasmid CRISPR-Cas9 system that can be delivered by both conjugation and electroporation was developed, and its efficiency was demonstrated by successfully targeting the *pyrE* locus. The CRISPR-Cas9 system was next used to target candidate genes encoding type IV pili, hypothesized by us to be involved in extracellular electron transfer (EET) in this organism. Single and double deletion strains (Δ*pilA*, Δ*pilE*, and Δ*pilAE*) were successfully generated. Additionally, the CRISPR-Cas9 tool was validated for constructing genomic insertions (Δ*pilAE::gfp* and Δ*pilAE::λ*_*Pr*_*gfp*). Finally, as type IV pili are believed to play an important role in extracellular electron transfer to solid surfaces, *C. metallidurans* CH34 Δ*pilAE* was further studied by means of cyclic voltammetry using disposable screen-printed carbon electrodes. Under these conditions, we demonstrated that *C. metallidurans* CH34 could generate extracellular currents; however, no difference in the intensity of the current peaks was found in the Δ*pilAE* double deletion strain when compared to the wild type. This finding suggests that the deleted type IV pili candidate genes are not involved in extracellular electron transfer under these conditions. Nevertheless, these experiments revealed the presence of different redox centers likely to be involved in both mediated electron transfer (MET) and direct electron transfer (DET), the first interpretation of extracellular electron transfer mechanisms in *C. metallidurans* CH34.

## Introduction

Gram-negative *Cupriavidus metallidurans* CH34 displays an extraordinary genomic plasticity^1^ that allowed it to evolve a unique combination of metabolic features, such as autotrophic growth on CO_2_ as the sole carbon source combined with hydrogenotrophy;^2,3^ degradation of BTEX (benzene, toluene, ethylbenzene, xylene);^3,4^ high resistance to numerous metals;^3^ biomineralization of gold,^5^ platinum,^6^ silver^7^ and uranium;^8^ and accumulation of polyhydroxybutyrate.^7^ However, despite this exceptional potential as an industrial microbial chassis for applications such as bioremediation, biomining, biosensing, carbon sequestration, and bioprocessing, proof-of-concept studies investigating this microorganism for biotechnological applications are scarce and mostly limited to its use in the bioremediation of metal-contaminated soils^9^ and waters.^10^

The major limitations hindering the study and use of *C. metallidurans* CH34 for biotechnological applications are the scarcity of genetic tools that can be adopted by the synthetic biology community to design genetic circuits of predicted outcomes, including the lack of fast marker-less genome editing tools, and of physiological studies unraveling industrially relevant features of *C. metallidurans* CH34 other than those linked to metal resistance. In fact, no genetic part libraries are currently available for *C. metallidurans* CH34. As such, developing and tailoring synthetic genetic circuits are difficult as knowledge of functional inducible and constitutive promoters with a diverse range of activity is lacking. Furthermore, genome editing in *C. metallidurans* CH34 has historically been performed by means of suicide plasmid-based allelic exchange technologies^11^ bolstered by counterselection and the transient expression of the *cre* recombinase to excise the antibiotic cassette.^11,12^ However, this process depends on multiple conjugation steps, which is not only a long and tedious process (e.g., when compared to electroporation^13^) but can also activate endogenous mobile genetic elements, leading to unwanted off-target modifications.^14^

Since their discovery, spCas9 systems have been extensively exploited as efficient and robust tools for the construction of recombinant prokaryotic^15^ and eukaryotic strains.^15,16^ Development of successful CRISPR-spCas9 technologies for selection of HR-mediated recombinant isolates in *Corynebacterium glutamicum*,^17^*Rhodobacter sphaeroides*,^18^*Methylococcus capsulatus*,^19^*Cupriavidus necator* H16,^20^ and *Clostridia* sp.^21^^21^ was shown to be dependent on two main factors. On the one hand, Cas9 expression needs to be tightly controlled via the use of inducible promoters^20^ and/or orthogonal synthetic riboswitches^21^ to prevent its toxicity. On the other hand, the use of strong constitutive promoters for the expression of single guide RNA (sgRNA) is required. However, as different microbes respond dramatically differently to regulatory sequences, host-dependent optimization of CRISPR-Cas systems is a prerequisite for their successful application.

The most recent proof-of-concept study focusing on potential biotechnological applications of *C. metallidurans* CH34 highlighted its capacity to degrade toluene while generating bioelectricity in microbial fuel cells (MFCs),^22^ thereby opening the possibility to use this microorganism for the remediation of wastewaters contaminated with recalcitrant xenobiotic and simultaneous recovery of energy. As current wastewater treatment technologies require more than 2% of the world’s electricity production (with local municipalities using up to 20% of their energy supply for recovery of wastewaters),^23^ development of efficient MFC technologies able to purify wastewaters and recover energy in the form of electricity is needed. Nevertheless, MFCs struggle to find commercial applications due to a low voltage output.^24^ To circumvent this issue, synthetic biology toolboxes, which encompass promoter libraries and CRISPR-Cas technologies, can be exploited to precisely tune and control extracellular electron transfer (EET) pathways, enhance the electron transfer rate between the electrode and the bacteria, and unravel EET mechanisms in different bacteria.^25^

The study of bacterial EET has been mainly focused on *Shewanella* spp.^26^ and *Geobacter sulfureducens*,^27^ two electroactive bacteria archetypical for mediated and direct external electron transfer, respectively. Of particular interest are the type IV conductive pili of *G. sulfureducens*,^27^ which are polymers of the PilA protein^28^ and have been postulated to be essential for the high anodic current generation in MFC with pure cultures of *G. sulfureducens*.^29^ PilA-based nanowires are therefore a promising sustainable conductive material that can be produced without the use of harsh chemicals while recovering energy and purifying wastewaters.^30^ In 2013, it was discovered that during the process of gold biomineralization, *C. metallidurans* CH34 formed biofilms on gold granules. Cells in the biofilm were shown to produce proteinaceous structures, which were hypothesized to be conductive nanowires used by the bacteria to “breathe” excess electrons outside the cell,^31^ thereby possibly expanding the list of bacteria employing type IV pili for EET.

In this study, we developed *C. metallidurans* CH34 as an industrial microbial chassis by (i) constructing a toolbox with constitutive and tightly controlled promoters, (ii) developing a single-plasmid CRISPR-Cas9 system deliverable by electroporation for marker-less editing and chromosomal DNA insertions, and (iii) demonstrating the use of this CRISPR-Cas9 system by exploring the involvement of the type IV pili in EET.

## Results and Discussion

### Optimization of Cas9 and sgRNA Transcription and the Design of the Single-Plasmid Genome Editing System

To successfully implement the CRISPR-Cas9 system for engineering *C. metallidurans* CH34, we aimed at (i) the transient expression and tight regulation of the toxic Cas9 protein, (ii) a high constitutive expression of the sgRNA, and (iii) the integration of the regulatory elements, the Cas9 coding sequence, a sgRNA and homologous template in an easy-to-cure plasmid platform.

**Figure 1:**
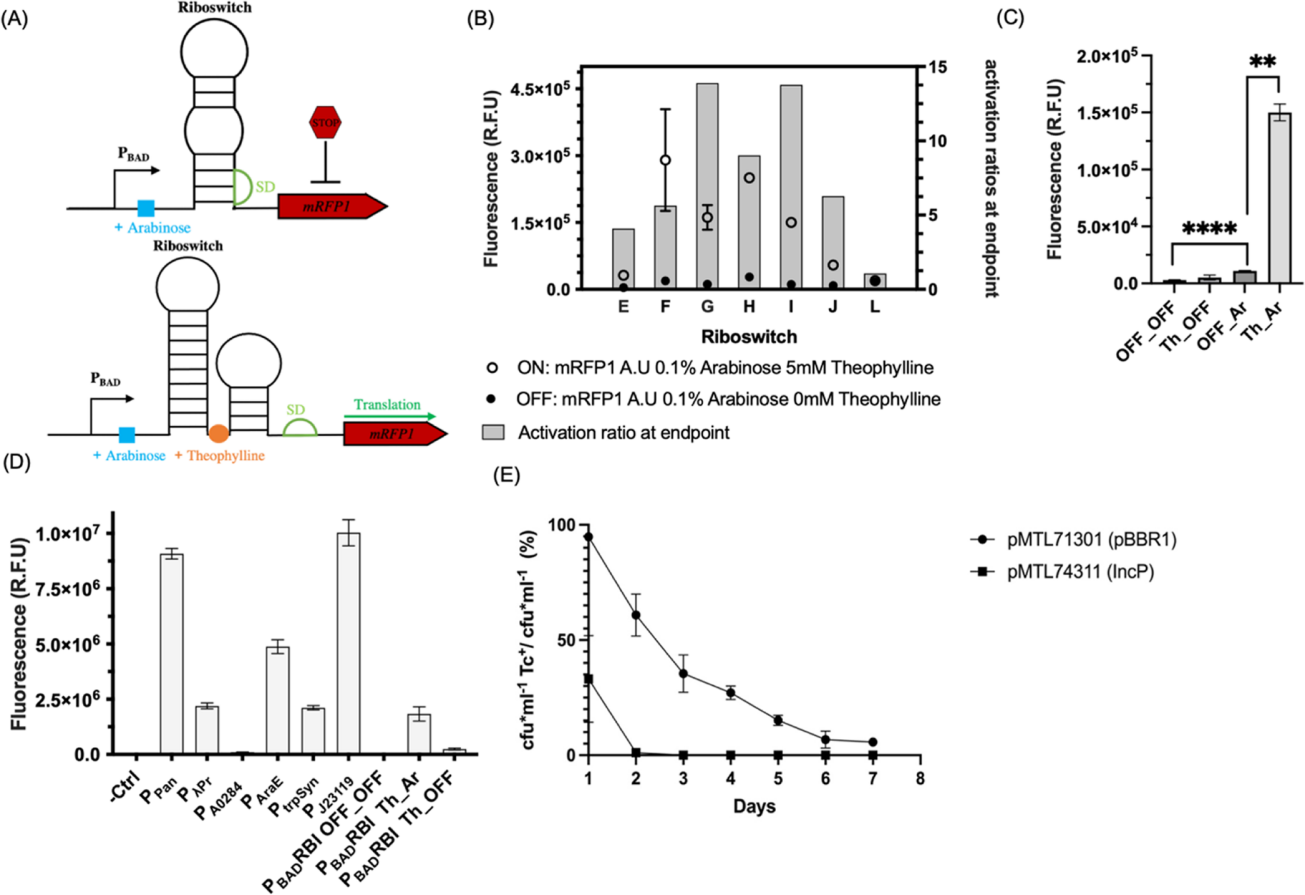
Optimization of the CRISPR-Cas9 system components, promoters, riboswitches, and plasmid backbones. (A) Schematic representation of the dual controlling mechanism using the arabinose inducible promoter (P_BAD_; transcriptional control) cloned upstream of a riboswitch library in the absence and presence of theophylline, a translational regulator that controls the translation of a modified red fluorescent protein (mRFP1, red). In the presence of arabinose and in the absence of theophylline (top panel), the SD (green) is not accessible for the translation of the mRFP1. The addition of theophylline (bottom panel, orange) results in the conformational change of the mRNA, making the SD available for mRFP1 translation. (B) Normalized activity of the P_BAD_-driven and theophylline-controlled riboswitch library (six riboswitch sequences labeled as E, F, G, H, I, J as previously described^23,38^ and L, control without a riboswitch sequence) cloned upstream the mRFP1 fluorescent protein. Fluorescence was measured in relative fluorescence units (RFU). The mRFP1 translation levels were measured in both the ON state (0.1% l-arabinose and 5 mM theophylline) and in the OFF state (0.1% l-arabinose only), and these are represented by empty and filled circles, respectively. The activation ratio (fluorescence difference between the ON and OFF states) at the endpoint is represented by gray bars. (C) Relative contribution of the RBI (riboswitch “I”, best performing as seen in (B)) in translational repression of the mRFP1 measured in RFU. The OFF_OFF state represents the RFU measured in the absence of both arabinose and theophylline (very low level of transcription and translation), the Th_OFF state represents the RFU measured in the absence of arabinose and the presence of theophylline, the OFF_Ar represents the RFU measured in the presence of arabinose and the absence of theophylline, while the Th_Ar represents the RFU measured in the presence of both arabinose and theophylline. (D) Normalized fluorescence of the mRFP1 fluorescent protein in *C. metallidurans* CH34 transformed with a multicopy plasmid (pMTL71301) carrying the mRFP1 cloned downstream of a constitutive promoter library (six promoter sequences labeled as P_Pan_, P_λPr_, P_A0284_, P_AraE_, P_trpSyn_, P_J23119_), measured alongside a negative control (−Ctrl, mRFP1 without a promoter sequence) and the previously characterized RBI (as seen in (C)). (E) Plasmid stability test of two replicative plasmids pMTL71301, carrying the pBBR1 origin of replication and pMTL74311 carrying the IncP origin of replication in *C. metallidurans* CH34. Both plasmids carry the tetracycline resistance gene, and the plasmid loss was determined after 1–7, 24 h serial passages in media without selection and as the ratio of colony forming units (cfu) after replica plating on agar plates with (cfu·mL^–1^ Tc^+^) and without (cfu·mL^–1^) tetracycline, as described previously.^42^ Error bars represent standard deviation (*n* = 4), and statistical significance was calculated by means of the Mann–Whitney test (**p < 0.00001, ****p < 0.001).

First, to achieve tightly regulated and transient expression of *cas9*, a two-level system was constructed based on the l-arabinose-inducible P_BAD_ promoter and a theophylline-dependent riboswitch library. These riboswitches were composed of a linker sequence, an aptamer domain specific for theophylline, and an expression domain harboring the Shine Dalgarno sequence (SD).^32^ When theophylline is absent from the growth media, the SD is occluded in the secondary structure formed by the transcribed riboswitch nucleotide sequence. However, upon ligation of theophylline to the aptamer domain, relaxation of the secondary structure occurs, and the SD becomes available for translation (Figure 1A). Theophylline-dependent riboswitches were previously successfully employed by Cañadas et al. for the controlled expression of *cas9* in *Clostridia* species.^21^ Here, we placed the P_BAD_ promoter upstream of the theophylline-dependent riboswitches F–J^32^ and E^21^ used by Cañadas and colleagues. A construct lacking any riboswitch (L, Linker) but maintaining the SD was used as a control. The plasmid series pMTL71301_P_BAD__RBX_*mRFP1* (where “X” represents the riboswitch under investigation) was transformed into *C. metallidurans* CH34 and mRFP1 expression scored by fluorescence intensity (au) (Table S8). The P_BAD_-riboswitch library was also tested in the presence of l-arabinose only (OFF state) to provide information regarding the ability of the riboswitch alone to control expression at the posttranscriptional level and in the presence of both l-arabinose and theophylline (ON state). The P_BAD__RBE, P_BAD__RBG, P_BAD__RBI, and P_BAD__RBJ constructs exhibited good repression of mRFP1 expression in the OFF state. However, P_BAD__RBE and P_BAD__RBJ were excluded from further consideration because they showed a low activation ratio, which is not ideal since suboptimal Cas9 expression levels may result in an inefficient selection of recombinant isolates.^33^ P_BAD__RBF was also excluded because of its high variability in terms of maximal mRFP1 expression levels recorded in the ON state (Figure 1B). Next, the relative contribution of the P_BAD_ and RBI elements was further investigated. The P_BAD__RBI system without both inducers (OFF_OFF) showed negligible mRFP1 expression. The addition of theophylline alone (Th_OFF) resulted in a small increase in expression (not statistically significant), which could be attributed to the background leakiness of the P_BAD_ promoter.^34^ However, while addition of only l-arabinose (OFF_Ara) resulted in a small but significant increase in mRFP1 production (Mann–Whitney test, *p* < 0.00001), the difference in mRFP1 expression between the OFF_Ara and Th_Ara states (with both inducers) was much more pronounced (Mann–Whitney test, *p* < 0.001), suggesting that the riboswitch effectively prevented translation by occluding the SD (Figure 1C). These results strongly indicate that the P_BAD__RBI combination could be used as a reliable ON/OFF system to ensure tight regulation of Cas9 levels.

To achieve a high constitutive expression of sgRNA in the CRISPR-Cas9 system, the P_Pan_,^35^ λ_Pr_,^36^ P_A0284_,^37^ P_AraE_,^21^ P_J23119_, and P_trpsyn_^38^^38^ constitutive promoters were cloned upstream of *mRFP1*, thereby generating the plasmid series pMTL71301_X_*mRFP1* where “X” stands for the specific constitutive promoter under study (Table S8). The plasmid series was transformed into *C. metallidurans* CH34 and mRFP1 expression scored by fluorescence intensity (au) (Figure 1D). While some promoters showed low to medium activity, P_Pan_ and P_J23119_ displayed strong constitutive expression (Figure 1D). P_J23119_ is a synthetic, fully characterized promoter of only 35 bp and much shorter than P_Pan_ (400 bp); therefore, it was selected for further work as it can more easily be cloned by designing primers with the promoter sequence as a spacer region (Table S9).

Since molecular tools for *C. metallidurans* CH34 are still very limited, the screening of constitutive and double inducible promoter libraries provides valuable information for the scientific community interested in using this species for biotechnological and synthetic biology applications. Promoters with low activities are useful for complementation studies (e.g., for auxotrophy)^39^ or the expression of toxic or difficult-to-purify proteins, i.e., to avoid the formation of inclusion bodies and improve the fraction of soluble protein.^40^ Moreover, leaking expression of a recombinant protein often leads to toxicity, growth deficiency, cell death, and loss of product. To this extent, dual expression control systems have been proposed as an efficient tool to suppress the leaky expression of proteins.^41^

Third, to find an easily curable plasmid as a backbone for the CRISPR-Cas9 system, a plasmid stability test using plasmid pMTL71301 (pBBR1 replication origin) and pMTL74311^42^ (IncP replication origin with single point mutation R271C in the *trfA* gene conferring high copy number^43^) was performed in *C. metallidurans* CH34, as previously described by Ehsaan et al.^42^ Plasmid pMTL74311 was completely lost after 48 h of continuous culture in the absence of selection, while plasmid pMTL71301 was still detectable in the cultures after 7 days (Figure 1E). For this reason, pMTL74311 was chosen as a suitable vector for the CRISPR-Cas9 system. In *C. necator* H16, the copy numbers per cell of pBBR1 and pCM271 (IncP *ori* with the R271C point mutation in *trfA*) were reported to be ∼40 for both replicons.^42^ Therefore, mRFP1 expression levels from plasmid pMTL74311 were assumed to be comparable to those quantified with the promoter libraries cloned in pBBR1 derivatives. A rapidly curable plasmid accelerates the isolation of plasmid-less mutant clones significantly.^44^ An alternative approach is the use of a temperature-sensitive plasmid backbone to achieve conditional plasmid propagation.^45^ However, such replicons cannot be used in *C. metallidurans* as it displays an aberrant phenotype at temperatures of 37–42 °C, which are required to induce plasmid loss.^3^

### Validation of the CRISPR-Cas9 System

**Figure 2:**
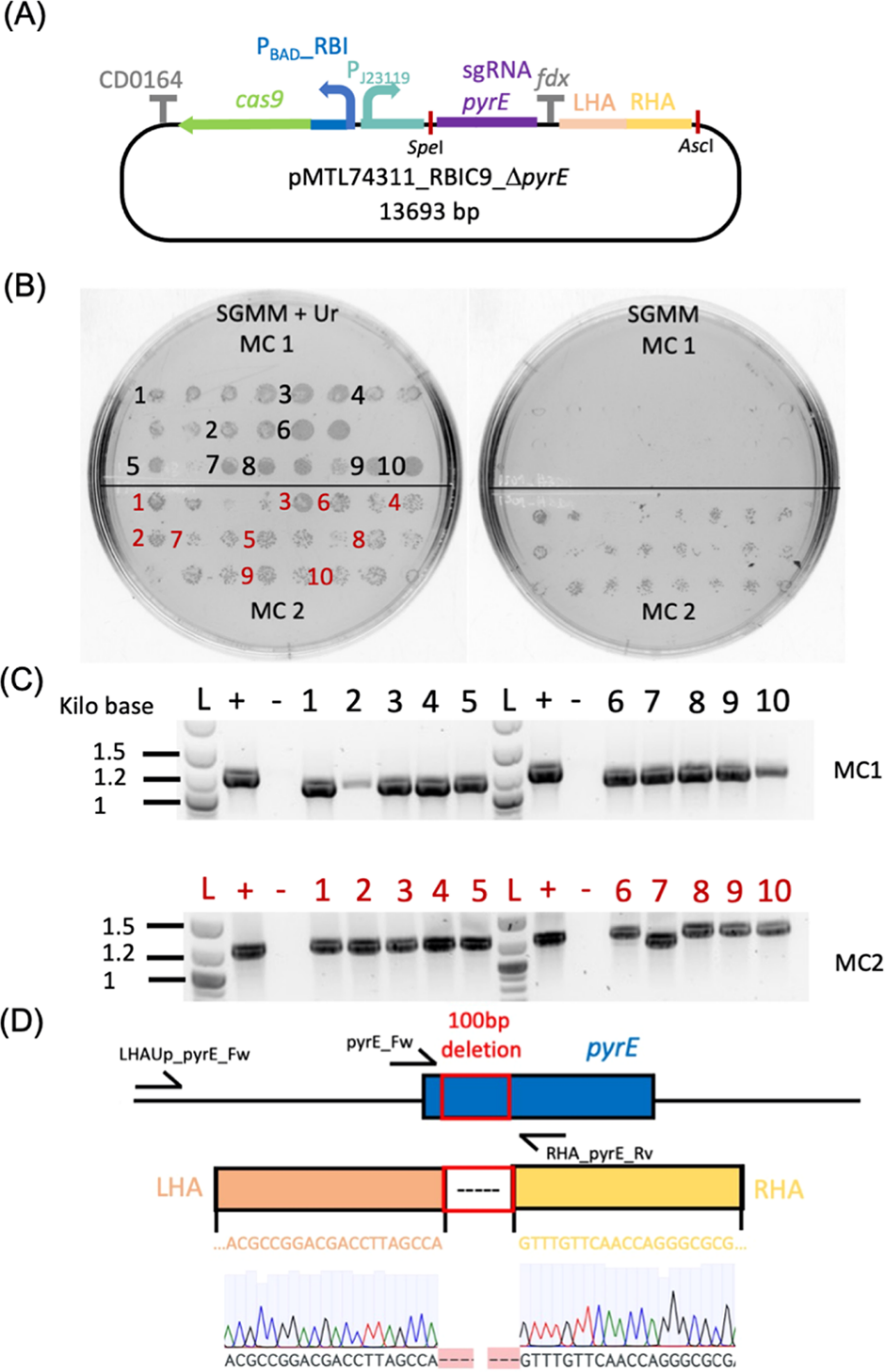
Isolation of *C. metallidurans* CH34 ΔpyrE deletion mutants using the optimized CRISPR-Cas9 system. (A) Graphical representation of the pMTL74311_P_BAD_RBIC9_ΔpyrE plasmid (13 693 kb) used to generate the pyrE deletion mutants. This plasmid carries two back-to-back transcriptional units and the homology regions (left homology Arm-LHA and right homology Arm-RHA) for homologous recombination. The first transcriptional unit is the cas9 driven by the P_Bad_, promoter and translationally regulated by the RBI riboswitch, terminated by the CD164 terminator sequence, the second is the small guide RNA (sgRNA) targeted to the pyrE region driven by the strong, constitutive, synthetic promoter P_j23119_, terminated by the ferredoxin (fdx) terminator sequence. The restriction sites *Spe*I and *Asc*I are highlighted. These can be used to modify the CRISPR plasmid for targeting the desired sequence. (B) Replica plating of two mother colonies (MC1 and MC2) on SGMM (sodium gluconate minimal medium) agar plates supplemented with uracil (+ Ur, left panel) or without uracil (right panels). Isolates selected for the following colony PCR are labeled in black and red for MC1 and MC2, respectively. (C) Agarose gel of amplicons obtained by PCR with primer pairs LHAUp_pyrE_Fw (binding outside the LHA)/pyrE_Rv on *C. metallidurans* CH34 colonies growing on SGMM agar plates. The expected amplicon sizes were ∼1.2 and 1.1 kb for the wild type and ΔpyrE knockout, respectively. For each gel, L, +, and – lanes were loaded with a DNA ladder and PCR products of the positive control (PCR amplicons from *C. metallidurans* CH34) and the negative control (PCR amplicon of pMTL74311_P_BAD_RBIC9_ΔpyrE), respectively. (D) Schematic representation of the pyrE locus reporting the pyrE gene (blue), the target deletion (red box), and the left and right homology arms (light orange and yellow, respectively) with the respective nucleotide sequences. The Sanger sequencing chromatogram of one of the isolates is also reported. Primer pyrE_Fw was used for sequencing the selected colonies.

Based on the data above, a CRISPR-Cas9 system was constructed based on the pMTL74311 (IncP) backbone with P_BAD__RBI-*cas9* and the J23119 promoter driving expression of the sgRNA. To assess if this CRISPR-Cas9 system was functional, we first constructed it with *pyrE*-specific sgRNA and HR arms to obtain a marker-free *pyrE* knockout in *C. metallidurans* CH34. The *pyrE* gene was selected as a target for the first evaluation because of the simplicity of screening for the Δ*pyrE* phenotype. Replica plating of bacterial colonies on minimal media agar plates with or without uracil can be used to quickly select for uracil auxotrophs, thereby allowing identification of recombinant clones, even in the case of low selection efficiency of Cas9. Replica plating of *C. metallidurans* CH34 isolates that appeared after theophylline and arabinose induction showed the successful isolation of recombinant mutants (Figure 2B shows a representative replica plating experiment). Randomly selected colonies screened by colony PCR and further confirmed via Sanger sequencing corroborated the successful isolation of *pyrE* knockouts, although recombination efficiency varied between each mother colony (MC) (Figure 2C,D). The overall efficiency of Δ*pyrE* mutant isolation was 60 ± 14.7% (*n* = 4, standard deviation), with selection efficiencies varying greatly between each experiment and each mother colony induced (Table 1). The evolution of bacterial cells is well known to cause the loss of performance in genetically modified bacterial strains due to mutation of recombinant DNA sequences,^46^ suggesting that “escapees” might be able to circumvent Cas9 selection, putatively by inactivating the portion of the *cas9* gene coding for its endonuclease domain or the promoter sequences controlling expression of *cas9* or the sgRNA. Nevertheless, the one mother colony that failed to produce knockouts did not show any growth on induction plates (MC3 from experiment number 2), suggesting that Cas9 selection may be, in some cases, 100% efficient in preventing the growth of nonrecombinant clones.

**Table 1:** Summary of the pyrE Knockout Experiment^a^

target	exp. number	MC ID	mutants/isolates screened	selection efficiency (%)	overall efficiency (%)
*pyrE*	1	1	12/12	54	60 ± 14.7
		2	1/12		
	2	3		100	
		4	12/12		
	3	5	2/12	58	
		6	12/12		
	4	7	3/12	29.1	
		8	4/12		

a Reported error represent standard deviation (*n* = 4, error is reported as the standard deviation).

Next, we targeted genes hypothesized to be involved in EET in *C. metallidurans* CH34. A bioinformatics analysis was carried out with the Uniprot BLAST tool to identify *G. sulfureducens* PilA (GSU1496) homologs in the proteome of *C. metallidurans* CH34. We found that in *C. metallidurans* CH34, Rmet_0472 and Rmet_0473 (annotated as type IVa pili PilA and PilE, respectively) were 49.1 and 42.4% identical to the full length of *G. sulfureducens* PilA. Moreover, in *G. sulflureducens*, GSU1496 and GSU1497 are found adjacent (similarly to *C. metallidurans* CH34) and functionally related to each other.^47^ Both GSU1496 and GSU1497 were found to be upregulated when *G. sulfureducens* was grown in MFC, and the GSU1497 knockout strain was also discovered to have the impaired capability of producing anodic currents.^47^ Therefore, given the genomic arrangement of Rmet_0472/Rmet_0473, the similarity of both genes with GSU1496 (Figure S1), the need for both GSU1496 and GSU1497, as well as the identical mapped peptidase cleavage site (G29) between Rmet_0472 and GSU1496, we hypothesized Rmet_0472 and Rmet_0473 to be involved in EET in *C. metallidurans* CH34. To test our hypothesis, plasmids pMTL74311_RBIC9_Δ*pilA*, pMTL74311_RBIC9_Δ*pilE*, and pMTL74311_RBIC9_Δ*pilAE* were constructed to generate knockouts of *pilA, pilE*, and *pilAE*, respectively. Plasmids pMTL74311_RBIC9_Δ*pilAE*::*gfp* (*gfp* expression controlled by a native promoter) and pMTL74311_RBIC9_Δ*pilAE*::λ_Pr_*gfp* (*gfp* expression controlled by a constitutive λ_Pr_ promoter) were also created to determine the ability of the CRISPR-Cas9 system to perform insertions, a necessary feature to develop recombinant strains for metabolic engineering. Plasmid pMTL74311_RBIC9_Δ*pilAE* was then delivered to *C. metallidurans* CH34 via conjugation. The MCs induced to target the *pilAE* locus resulted in a 100% editing efficiency. However, recombination of isolates from induction of MC2 resulted in the deletion of about 300 bp in the LHA (Figure S2 and Table S3). Electroporation was also investigated for plasmid delivery as conjugation has been reported to induce the SOS response genes, which could trigger DNA recombination,^48^ thereby reducing the chances of obtaining isogenic mutants. Electroporation would also save a considerable amount of time (∼2 days), particularly when employing a quick protocol for the preparation of electrocompetent cells. Furthermore, conjugation can also trigger endogenous mobile genetic elements creating unwanted off-target modifications.^49^ When plasmids were delivered by electroporation to target the *pilA* and *pilE* loci, the efficiency of selection was 12.5% for both targets. Genomic integration of the *gfp* gene with the concomitant deletion of the *pilAE* operon was also successfully achieved with an overall efficiency of 50%. However, no GFP expression was detected using super-resolution confocal microscopy. In *Pseudomonas aeruginosa*, the presence of the PilA protein was suggested to be required for its own regulation as a complex interaction between the PilSA two-component system results in the autoregulation of *pilA* expression.^50^ Thereby, it is possible that a similar regulation of the expression of the *pilA/E* genes may also occur in *C. metallidurans* CH34 and thus the deletion of the *pilAE* locus may have impaired expression from their native promoter, resulting in no production of GFP.

**Figure 3:**
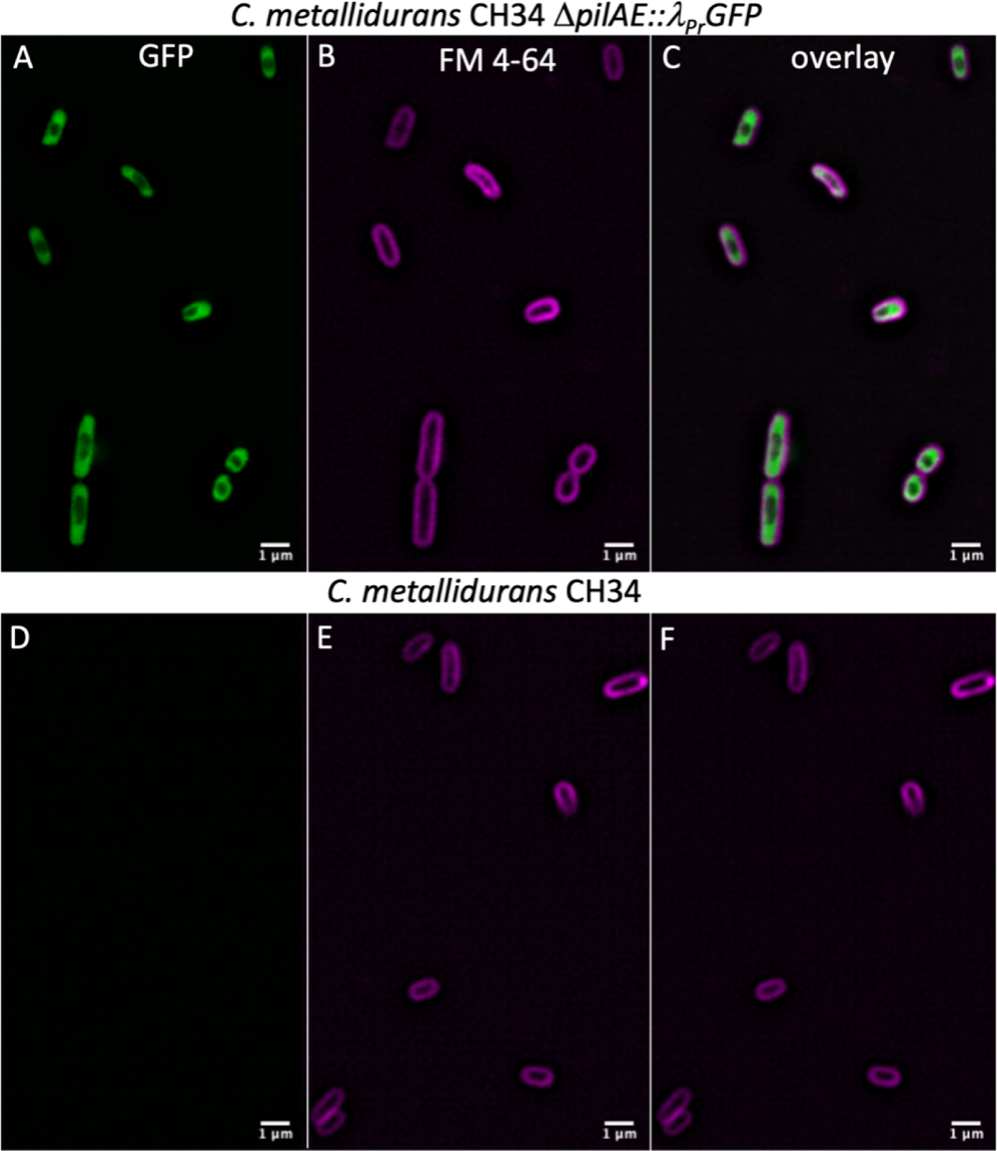
Localization of constitutively expressed GFP protein integrated into the genome of *C. metallidurans* by structural illumination microscopy (SIM). Panels (A)–(C) represent images of *C. metallidurans* CH34 Δ*pilAE::λ*_*Pr*_*GFP* expressing GFP intracellularly (A, bandpass filters BP 495–550 + LP 750), the cell membrane stained with FM-64 FX membrane dye (B, bandpass filter LP 655), and the overlayed channels (C). Panels (D)–(F) represent the wild-type *C. metallidurans* CH34 control strain, imaged under the same conditions as above.

Next, the insertion of *gfp* under control of the constitutive λ_Pr_ promoter was tested. Electroporation of plasmid pMTL74311_P_BAD_RBIC9_Δ*pilAE*::λ_Pr_*GFP* resulted in low transformation efficiency. Moreover, only 4 out of the 10 induced mother colonies produced clones on LB IND plates, none of which carried the desired mutation. The same plasmid was also delivered by conjugation, with an ∼100-fold increase in transformation efficiency (Figure S4). Two mother colonies were induced, and a total of 2 out of 93 isolates were confirmed by cPCR (colony PCR) to be Δ*pilAE*::λ_Pr_*GFP* mutants (2.03% efficiency). Therefore, although electroporation can be used, conjugation may improve editing efficiency for more difficult targets (e.g., for the integration of CDSs coding toxic proteins or in case of low electroporation efficiency). *C. metallidurans* CH34 Δ*pilAE*::λ_Pr_*GFP* was visualized under structural illumination microscopy. The wild-type strain was used to measure basal fluorescence levels, which were used as a reference for comparison with *C. metallidurans* CH34 Δ*pilAE*::λ_Pr_*GFP* (Figure 3). All knockouts were confirmed and successfully cured of the editing CRISPR-Cas9 plasmid by overnight growth in nonselective media followed by replica plating and cPCR with primers amplifying *cas9*. Finally, whole-genome sequencing of the knockout strains showed that only one of the mutants carried off-target mutations (Table S5). Results of the validation knockout experiments in terms of Cas9-mediated selection efficiencies are presented in Table 2, while representative agarose gel electrophoresis pictures of the cPCR products confirming *pilE* and *pilA, pilAE::GFP*, and *pilAE::*λ_Pr_*GFP* knockouts are reported in Figure S2.

**Table 2:** Summary of the Validation Experiment of the CRISPR-Cas9 System

target	delivery	mutants/screened	selection efficiency* (%)
*pilAE*	conjugation	16/16	100
*pilA*	electroporation	3/24	12.5
*pilE*	electroporation	3/24	12.5
*pilAE::GFP*	electroporation	12/24	50
*pilAE::*λ_Pr_*GFP*	electroporation	0/24	0
*pilAE::*λ_Pr_*GFP*	conjugation	2/93	2.04

### Use of Cyclic Voltammetry to Study the Involvement of Type IV Pili in EET of a *C. metallidurans* CH34 Biofilm on Screen-Printed Carbon Electrodes

**Figure 4:**
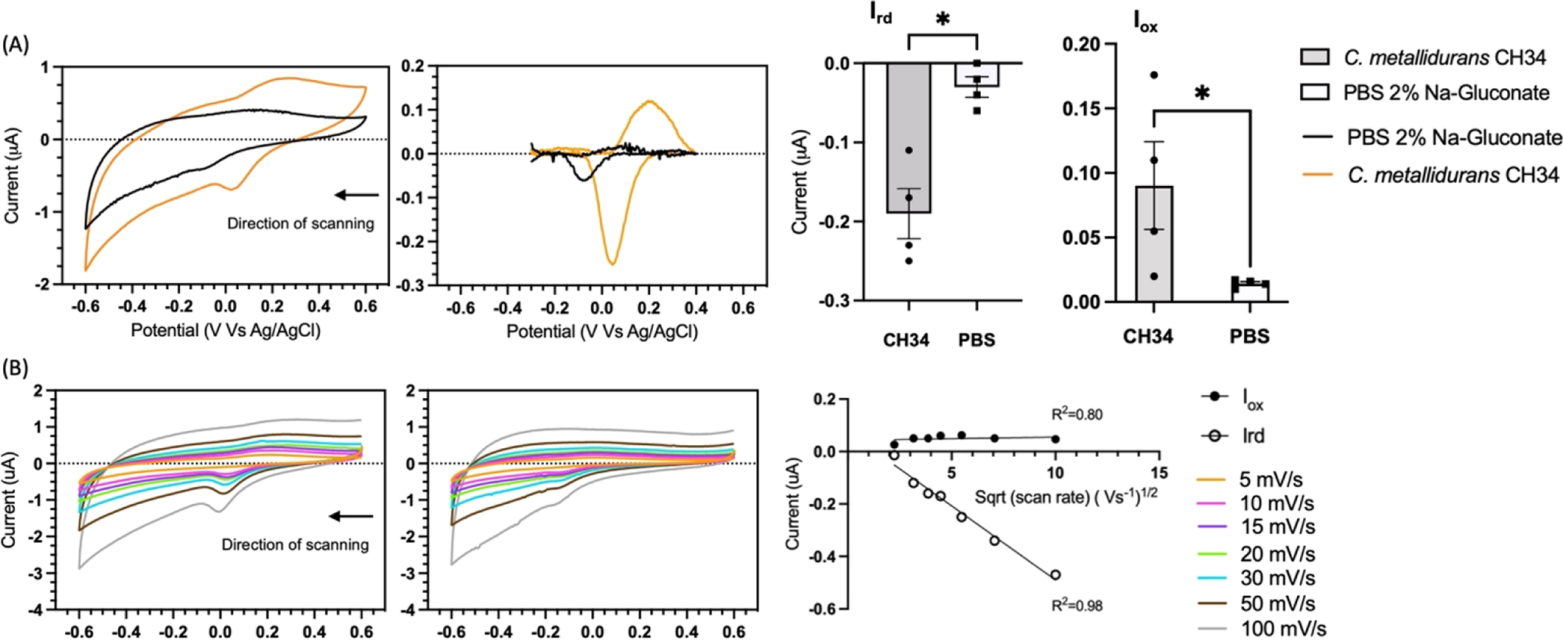
Cyclic voltammetry experiments of *C. metallidurans* CH34 grown on SPCEs and negative control. (A) Cyclic voltammetry at 20 mV/s. From left to right: (i) graph showing representative cyclic voltammetry of the biofilm-coated SPCE (orange voltammograms) and negative control (black voltammograms), (ii) voltammograms after baseline subtraction, and (iii) bar diagrams of the reduction and oxidation current peaks (*I*_rd_ and *I*_ox_, respectively) of the SPCE modified with *C. metallidurans* CH34 (gray bars) and negative control (white bars) are shown from left to right. (B) Scan rate experiment between 5 and 100 mV/s. From left to right: (i) graphs of the scan rate experiments of the biofilm-coated SPCE, (ii) negative control, and (iii) plot of the peak currents against the square root of the scan rate are reported, respectively, from left to right. Error bars represent the standard deviation (*n* = 4), and statistical significance was calculated by means of the Mann–Whitney test (*p < 0.05).

To confirm the successful colonization of the screen-printed carbon electrodes (SPCEs) by *C. metallidurans* CH34, these were subjected to live/dead staining after each cyclic voltammetry experiment and imaged using confocal microscopy. Bare electrodes incubated in the absence of bacterial cultures were used as a negative control in these experiments. Horizontal sections and Z-stacks of the biofilm-colonized electrodes showed the successful formation of a biofilm monolayer with most of the cells being alive, indicating that current peaks in the biofilm-modified electrodes resulted from the electroactivity of *C. metallidurans* CH34 (Figure S6). Cyclic voltammograms of the biofilm-coated SPCEs and negative controls were recorded at 20 mV/s. The biofilm-coated SPCEs showed a cathodic (reduction) and an anodic (oxidative) peak at ∼0 and 0.3 V. The negative control showed two smaller redox peaks at −0.1 V (reduction) and 0.1 V (oxidation) (Figure 4A left and mid panels represent the voltammograms as recorded and the same curves after baseline subtraction, respectively, between −0.4 and 0.4 V). The current peaks observed in the biofilm-modified SPCE were significantly higher than in the negative control (Mann–Whitney test *n* = 4, *p* < 0.05), thereby confirming the presence of redox centers involved in EET in *C. metallidurans* CH34 (Figure 4A, right panel). Next, to characterize the electron transfer behavior further, a scan rate experiment was performed. The redox couple observed at 20 mV/s could be observed at all scan rates for the biofilm-coated SPCEs, but not for the negative control (Figure 4B left and mid panels, respectively). The linear relationship between the cathodic peak and the square root of the scan rate suggested a diffusion-controlled EET system (Figure 4B, right panel). However, the anodic current peaks showed no linear relationship with either the scan rate or its square root and displayed reduced intensity when compared to the cathodic current peak (Figure 4B right panel and Figure S7 top panel). Furthermore, shifting of the cathodic and anodic peaks toward more negative and positive potentials, respectively, was also observed (Figure S7 bottom panel), which was previously reported to be due to a sluggish charge transfer rate and solution resistance.^51^ These observations suggested that the observed phenomenon was of quasi reversible ErCi (reversible electron transfer followed by an irreversible homogeneous chemical reaction) of diffusive nature.^52^ Cyclic voltammetry experiments of *G. sulfureducens* in MFC under acetate-oxidizing conditions showed anodic currents arising from surface-adsorbed and diffusion-limited mechanisms at different scan rate regimes. This complex interaction resulting in a “bimodal behavior” was discussed to be due to redox molecules confined in the biofilm.^53^ This electrochemical response is typical of well-established and thick biofilms of *Geobacter* and shows similarities to the catalytic response of glucose oxidase enzymes trapped in a redox conductive epoxy cement, which displays symmetric redox current peaks at slow scan rates typical of adsorbed species but exhibits classic diffusion-limited behavior at high scan rates.^54^ However, the *C. metalliduranas* CH34 biofilm showed the presence of a monolayer of cells (z-stack Figure S7), excluding the complex interplay of redox molecules trapped in thick biofilms and limiting data interpretation to the presence of soluble redox mediators of an E_r_C_i_ system.

**Figure 5:**
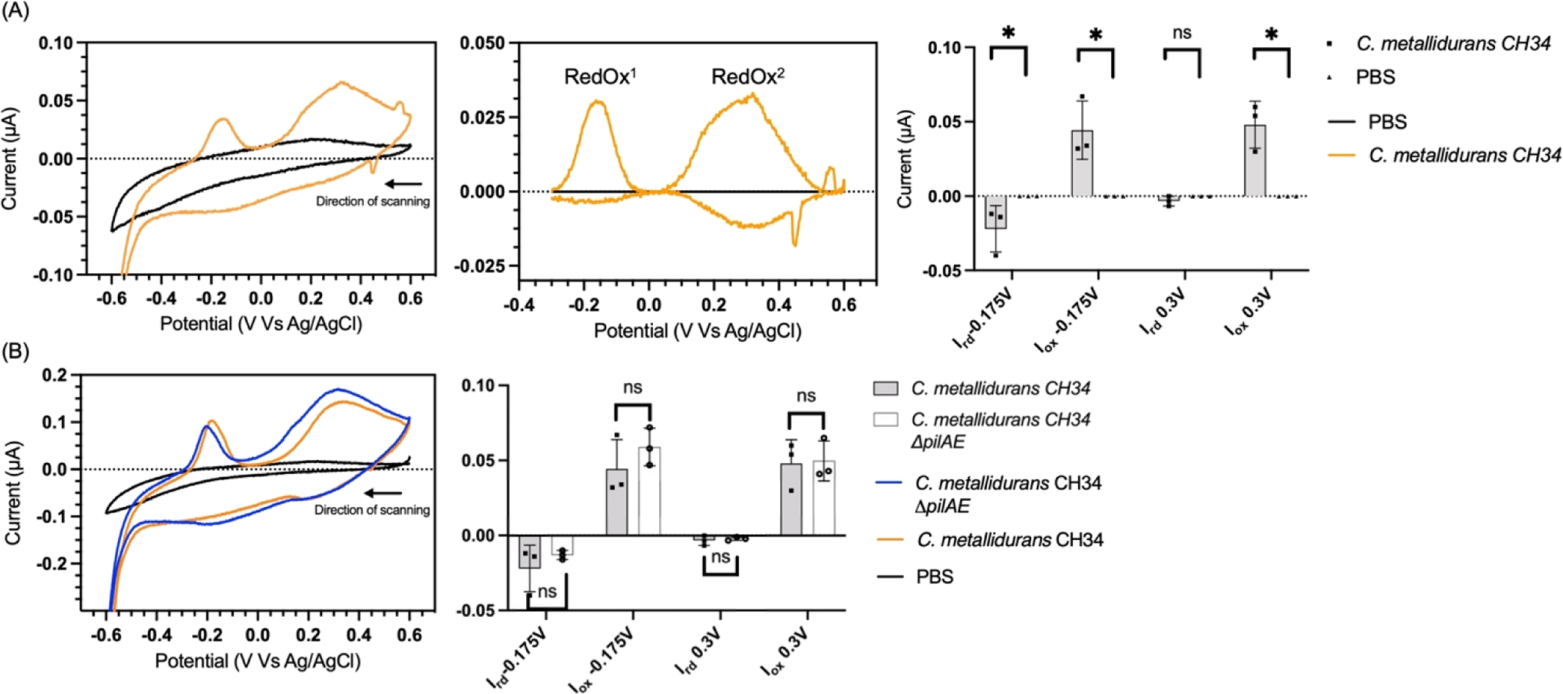
Cyclic voltammetry experiments at 1 mV/s of *C. metallidurans* CH34 and *C. metallidurans* CH34 ΔpilAE biofilms on SPCEs. (A) Cyclic voltammetry experiments of the SPCE modified with biofilms of *C. metallidurans* CH34 (orange tracks) and SPCE negative control (black tracks). From left to right: (i) panel of a representative voltammogram, (ii) current peaks after baseline subtraction, and (iii) bar diagrams of the current peaks of redox^1^ and redox^2^ are reported, respectively, from left to right. (B) Cyclic voltammetry experiment of the SPCE modified with biofilms of *C. metallidurans* CH34 (orange), *C. metallidurans* CH34 ΔpilAE (blue), and SPCE incubated without the cell cultures (black tracks). The left panel shows a representative voltammogram. The right panel displays a bar diagram of the oxidation and reduction current peaks of *C. metallidurans* CH34 and *C. metallidurans* ΔpilAE. Error bars represent the standard deviation (*n* = 3), and statistical significance was calculated by means of the Mann–Whitney test (*p < 0.05).

Next, to resolve potential redox peaks with low electrochemical activity, CV experiments were repeated at 1 mV/s. Two redox couples (redox^1^ and redox^2^) were observed (Figure 5A, left and mid panels represent the voltammogram as recorded and the same curves after baseline subtraction, respectively) with peak potentials of 0.3 and −0.16 V, respectively, which could not be observed in the negative control (Figure 5A, right panel). Both centers showed no peak separation and a ratio *I*_Rd_/*I*_Ox_ ≠ 1, thereby indicating two electrochemically active species adsorbed on the electrode surface to be responsible for EET in *C. metallidurans* CH34. To study the putative involvement of type IV pili in the EET process, *C. metallidurans* CH34 Δ*pilAE* was studied by CV at 1 mV/s. Neither differences in the shape of the voltammogram nor in the current intensity were observed between the parental strain and the Δ*pilAE* mutant, indicating that type IV pili are not involved in EET under the conditions tested (Figure 5B left and right panels, respectively). This null finding is important as the variables studied in this work inform on the role of electron transfer via conductive pili in *C. metallidurans* CH34, which has not been observed before but has only been hypothesized in a previous publication.^31^ Altogether, these results showed that *C. metallidurans* CH34 is capable of EET by means, possibly in a similar fashion to *Shewanella* MR1, which uses a set of periplasmic^55^ and outer membrane^56^ cytochromes and soluble electron carriers^57^ for electron storage and release to terminal electron acceptors.^58^

## Conclusions

In this study, a CRISPR-Cas9 tool for genome editing of *C. metallidurans* CH34 was developed. Preliminary steps included optimizing the expression level of the sgRNA and Cas9, pivotal to minimizing the toxicity of Cas9 and improving the overall efficiency of the system. Constitutive promoters with a wide range of activity and a hybrid ON/OFF system composed of the inducible P_BAD_ and ncRNAs riboswitches for complete repression of expression in the absence of the inducers were characterized and optimized. This is the first comprehensive library of constitutive and inducible regulative elements for *C. metallidurans* CH34, applicable much broader than the scope of this work. Our developed CRISPR-Cas9 system showed to be robust with an isolation efficiency of recombinant clones ranging between 2 and 100%.

In addition, the time-honored electrochemistry of cyclic voltammetry was applied to examine electron exchange mechanisms between the electrode surface and *C. metallidurans* biofilms. A comparison of the *C. metallidurans* CH34 parental and Δ*pilAE* strain did not show evidence of the involvement of type IV pili in the transfer of electrons under the conditions tested. Nevertheless, it did reveal the presence of different redox centers likely to be involved in both MET and DET. Two of these centers are likely to be outer membrane cytochromes forming direct contact between the cell and the electrode surface or polymers involved in electron storage and release. These data represent a first suggestion of the mechanisms *C. metallidurans* CH34 uses for extracellular electron transfer and shed light on the intricate multicomponent systems likely to be composed of cytochromes and diffusive mediators, which carry out oxidation and reduction under the limitation of a carbon source and a terminal electron acceptor.^59^

## Materials and Methods

### Bacterial Strains and General Growth Conditions

The general growth of *C. metallidurans* CH34 and derivative strains was performed in a lysogeny broth (LB) at 30 °C. *Escherichia coli* was grown in LB at 37 °C (shaking at 220 rpm was added as a condition for liquid cultures). When necessary, 20 μg/mL tetracycline was used for both *C. metallidurans* CH34 and *E. coli* strains (Tc^+^, Gen^+^). Gentamycin at a concentration of 10 μg/mL was used when growing *C. metallidurans* CH34 strains.

### Plasmid Design and the Cloning Procedure

All of the plasmids used in this study are reported in Table S8. Oligonucleotide primers were synthesized by IDT DNA Technologies, and their sequences are reported in Table S9. The P_Pan_ promoter was synthesized with no codon optimization by Genescript Biotech (Leiden, The Netherlands) and was delivered in the plasmid pUC57_P_pan_.

A Q5 High-Fidelity 2X Master Mix (NEB) and a QuickLoad Taq 2X master mix were used for PCR amplification for cloning and colony PCR, respectively. An NEB Builder Hi-Fi DNA Assembly Master Mix (NEB) was used for the assembly of plasmid DNA after the plasmid design was carried out using the NEBuilder tool. A QIAprep Spin Miniprep Kit was used to isolate plasmid DNA (Qiagen). Restriction enzymes were purchased from NEB, and gel-purified DNA fragments were extracted with a Zymoclean Gel DNA Recovery Kit (Zymo Research) as per the manufacturer’s recommendations. Plasmids were confirmed by Sanger sequencing (Eurofins). Details of plasmid construction are reported in the Supporting Information. Chemically competent *E. coli* DH5a and *E. coli* S17-1 were transformed via heat shock with the plasmid of interest.^60^

### Plasmid Delivery in *C. metallidurans* CH34

Bacterial conjugation was performed by growing overnight in liquid LB media. *C. metallidurans* CH34 and *E. coli* S17-1 were transformed with the desired plasmid. The following day, 1 mL of each culture was washed twice in PBS and eventually resuspended in 30 μL of PBS. The cultures were then mixed, placed on an LB–agar Petri dish (Starstedt), and incubated overnight at 30 °C. Plasmids pMTL74311_RBIC9_Δ*pyrE* and pMTL74311_RBIC9_Δ*pilAE* were delivered via conjugation. pMTL74311_RBIC9_Δ*pilE*, pMTL74311_RBIC9_Δ*pilA*, and pMTL74311_RBIC9_Δ*pilAE::GFP* were delivered via electroporation using a protocol described elsewhere for the preparation of the competent cells and electroporation.^61^ Plasmid pMTL74311_RBIC9_Δ*pilAE::λ*_*Pr*_*GFP* was delivered via both conjugation and electroporation.

### Study of the Promoter and Riboswitch Libraries

*C. metallidurans* CH34 was electroporated with the plasmids for the riboswitch or constitutive promoter libraries. Cultures were resuspended to OD_600_ 0.05 in fresh LB media, and 200 μL was distributed in a black bottom 96-well plate (Thermo Scientific). The plates were incubated in a Cytomat 2C incubator at 30 °C, 800 rpm for 48 h, and were fed into a Molecular Devices SpectraMax i3 plate reader using a PreciseSCARA robotic arm OD_600_. mRFP1 was excited at 585 nm, emission was detected at 620 nm, and OD measurement was done at 600 nm. mRFP1 levels (Arbitrary Units, au) at the end of the experiment were plotted for each plasmid construct for the constitutive promoter and riboswitch library. For the P_BAD__Riboswitch library, mRFP1 expression levels were studied in the presence of 5 mM theophylline and 0.1% l-arabinose (ON_ON) or in the presence of l-arabinose only (OFF_ON). Activation ratios were calculated as follows
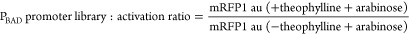
A study of the relative contribution of the P_BAD_ promoter and RBI to the expression of mRFP1 was performed without inducers (OFF_OFF), in the presence of only theophylline (Th_OFF), only arabinose (OFF_Ar), and both inducers (Th_Ar)

### Protocol for the Validation of the CRISPR-Cas9 System

To obtain knockouts of *C. metallidurans* CH34, the procedure was adapted and modified from the work of Cañadas and colleagues^21^ as follows.

#### Selection of Transformants and the Isolation of Mother Colonies

After 3–5 days of the delivery of the plasmid, transformants were selected on LB Tc^+^ Gen^+^ agar plates. Two colonies for each transformation event were streaked on LB Tc^+^ Gen^+^ agar plates to generate mother colonies with enough biomass for induction.

#### Induction of the Mother Colonies

A 1 μL loop was used to resuspend the mother colonies in 100 μL of PBS. These were then plated on LB induction agar plates (LB IND, supplemented with tetracycline, gentamycin, 5 mM theophylline, 0.1% l-arabinose) and incubated at 30 °C for 3–5 days.

#### Confirmation of Recombinant Isolates

Isolates growing on the LB induction agar plates were then streaked on LB Gen^+^ agar plates and subjected to colony PCR with primer pair external to the homology arms and primer pairs with one primer external to the homology arms and the other primer inside the deleted genes.

#### Curing of CRISPR-Cas9 Plasmids

Recombinant clones were grown overnight in LB Gen^+^ at 30 °C 200 rpm, serially diluted, plated on LB Gen^+^ agar plates, and incubated at 30 °C for 24 h. Overall, 48 isolates were resuspended in 100 μL of PBS and replica plated on LB Tc^+^ Gen^+^ and LB Gen^+^ to verify the plasmid loss. Finally, cPCR with primer pairs C9_cPCR_Fw/Rv amplifying Cas9 (Table S9) was performed to exclude the presence of the editing plasmid.

#### Storage of the Recombinant Isolates

cPCR was performed again on isolates that lost the plasmid (not growing on LB Tc^+^ Gen^+^ agar plates). These were then grown overnight in LB Gen+ for the creation of cell banking.

To obtain the Δ*pyrE* knockouts, the following modifications were made to the protocol.

#### Selection of Transformants and the Isolation of Mother Colonies

Selection of transformants was performed on SGMM Tc^+^ Gen^+^ Ur^+^ agar plates.

#### Induction of the Mother Colonies

Induction of the mother colonies was performed on SGMM Tc^+^ Gen^+^ Ur^+^ Th^+^ Ar^+^ agar plates.

#### Confirmation of Recombinant Isolates

Confirmation of recombinant isolates was performed on SGMM Gen^+^ Ur^+^ agar plates.

#### Curing of CRISPR-Cas9 Plasmids

Recombinant clones were grown overnight in SGMM Gen^+^ Ur^+^ at 30 °C 200 rpm, serially diluted, plated on SGMM Gen^+^ Ur^+^ agar plates, and incubated at 30 °C for 24–48 h. Overall, 48 isolates were resuspended in 100 μL of PBS and replica plated on SGMM Gen^+^ Tc ^+^ Ur^+^ and SGMM Gen^+^ Ur^+^ to verify the plasmid loss.

#### Storage of the Recombinant Isolates

This was performed as for other recombinant strains, but the growth of isolates was done using SGMM Gen^+^ Ur^+^ liquid media.

### Growth of the Biofilm of *C. metallidurans* sp. on SPCE

*C. metallidurans* CH34 was grown in 7.5 mL of SGMM in a 50 mL falcon tube, shaking at 220 rpm and 40 °C for 48 h. It was then washed twice in PBS and resuspended in PBS pH 6.9 at OD_600_ 3.5. A total of 4 mL of the culture was placed into a 20 mL scintillation vial (Starstedt) and transferred into an anaerobic cabinet (Don Whitley Scientific). SPCE was washed with ddH_2_O, sterilized with 70% ethanol, rinsed again in ddH_2_O, transferred to the scintillation vial, and incubated for 24 h in AnO_2_ conditions. After incubation, the electrode was gently rinsed with PBS pH 6.9 2% Na–gluconate. Then, 200 μL of PBS pH 6.9 2% Na–gluconate was then placed so to cover the electrodes, and cyclic voltammetry experiments were finally performed.

### Confocal Microscopy

*C. metallidurans* CH34, *C. metallidurans* CH34 Δ*pilAE::GF*, and *C. metallidurans* CH34 Δ*pilAE::λ*_Pr_*GFP* were grown as previously described. Bacterial cultures were normalized to OD_600_ 0.5 and collected by centrifugation at 4000 rpm for 5 min. After two washes with PBS, cell membranes were stained with 5 mM FM 4-64 (ThermoFisher) dye in PBS for 30 min in the dark. After staining, cells were collected by centrifugation at 2000 rpm for 10 min, washed twice with PBS, and eventually resuspended in 100 mL of a Fluoromount-G water-based mounting medium (ThermoFisher). Overall, 5 μL of a cell suspension was placed on an 18 mm borosilicate glass square coverslip (FisherScientific) and mounted on a frosted microscope slide (Thermo Scientific). Images were taken with a Zeiss Elyra Super Resolution Microscope using a Plan-Apochromat 63×/1.4 Oil DIC M27 objective. The structured illumination microscopy (SIM) mode was used with the following settings. Two tracks were set up for lasers 488 nm at 4 and 8% power and 100 and 200 ms exposure for GFP and FM 4-64, respectively. Super-resolution microscopy (SRM) and SIM grating periods were 28 and 42 mm for the green and red tracks, respectively, while bandpass filters employed were BP 495–550 + LP 750 for the green channel and LP 655 for the red channel. The channel shift was corrected using the Zen Channel Alignment Tool.

For live/dead staining of the SPCEs, after cyclic voltammetry experiments, the SPCE used for the study of electrophysiological properties of *C. metallidurans* CH34 was removed from AnO_2_ conditions and stained for 30 min in the dark at RT with 5 mM Syto9 and 20 mM PI. After staining, the electrodes with the staining solution were placed in 35 mm Nunc Glass Bottom Dishes (ThermoFisher) and imaged using a Zeiss Elyra Super Resolution Microscope using a Plan-Apochromat 20× objective. The excitation of Syto9 and PI was performed with lasers 488 nm and 561 nm, while the detection was obtained in the windows of 499–570 and 588–685, respectively. The power of both lasers was set at 1%. The Z-stack was obtained by scanning 17 slices of 16 μm each.

### Cyclic Voltammetry

Zensor TE-100 SPCE was used for all of the electrochemical analysis. Initially, an electrode batch test (*n* = 5) was performed by covering WE, CE, and RE with 1 mM ferricyanide in AnO_2_ conditions in an anaerobic cabinet (Don Whitley Scientific) with an mStat-i 400s portable potentiostat (Metrohm DropSens). The pretreatment of the electrodes was performed by repetitive cycling by performing CV between 0.6 and −0.6 V at 100 mV/s vs Ag/AgCl for 70 cycles.

Electrochemical experiments were all performed in AnO_2_ conditions. *C. metallidurans* CH34 was grown for 48 h in 7.5 mL of SGMM in a 50 mL falcon tube, washed twice in PBS, and resuspended in PBS pH 6.9 at OD_600_ 3.5. A total of 4 mL of the culture was placed in a 20 mL scintillation vial (Starstedt) and transferred into an AnO_2_ cabinet (Don Whitley Scientific). CV measurements of biofilm-modified SPCEs were taken by performing CV between 0.6 and −0.6 V at 1, 5, or 20 mV/s vs Ag/AgCl. For scan rate studies, CV experiments were performed at 5, 10, 15, 20, 30, 50, and 100 mV/s vs Ag/AgCl. SPCE incubated in PBS pH 6.9 but with no cells was used as a negative control.

Dropview 8400 software (Metrohm DropSens) was used for recording voltammograms. A polynomial fitting baseline subtraction tool was used for the subtraction of the baseline from current peaks.

### Bioinformatic and Statistical Analysis

The Uniprot Blast tool (https://www.uniprot.org/blast) was used to identify similar primary amino acid sequences of *G. sulfureducens* PilA (GSU1496) in the genome of *C. metallidurans* (strain ATCC 43123/DSM 2839/NBRC 102507/CH34). Clustal Omega (https://www.ebi.ac.uk/Tools/msa/clustalo/) was used to align the amino acid sequences between type IV pili of *G. sulfureducens* and *C. metallidurans* CH34. Illumina sequencing was provided by MicrobesNG (https://microbesng.com). CLC Genomic Workbench was used to analyze the whole-genome sequencing data. Program default parameters were used to analyze single nucleotide variants (SNVs), insertion and deletions (InDels), and structural variations (SVs).

## Supporting Information Available

Plasmid construction, (Figure S1) clustal alignment, (Figure S2) and (Table S3) colony PCR strategy, (Figure S4) transformation efficiencies of pMTL74311_P_BAD_RBIC9_Δ*pilAE*::λ*GFP*, (Table S5) illumina sequencing of *C. metallidurans* CH34 knockouts, (Figure S6) confocal microscopy of biofilms, (Figure S7) scan rate experiment, (Table S8) plasmids and strains, (Table S9) oligonucleotide sequences, and (Table S10) spacer sequences (PDF)

## Abbreviations

cPCR: colony PCR
CDS: coding sequence
EET: extracellular electron transfer
MET: mediated electron transfer
MFC: microbial fuel cells
SD: Shine Dalgarno sequence
SIM: structural illumination microscopy
SPCE: screen-printed carbon electrodes
