# Supplementary material for: Synthetic Biology Toolbox, Including a Single-Plasmid CRISPR-Cas9 System to Biologically Engineer the Electrogenic, Metal-Resistant Bacterium Cupriavidus metallidurans CH34
Source: ACS Synth Biol. 2022 Oct 24;11(11):3617–28. doi: 10.1021/acssynbio.2c00130 (PMC9680026; doi:10.1021/acssynbio.2c00130)
Supplement: Supplementary file 1 — sb2c00130_si_001.pdf [file sb2c00130_si_001.pdf]

Supporting information

A Synthetic Biology Toolbox, Including a Single-Plasmid CRISPR-Cas9 System for Biological Engineering the Electrogenic, Metal-Resistant Bacterium *Cupriavidus metallidurans* CH34

Federico Turco[a], Marco Garavaglia[b], Rob Van Houdt[c], Phil Hill[d], Frankie J. Rawson[a] and Katalin Kovacs[e,*]

[a]Federico Turco - School of Pharmacy, BioDiscovery Institute, University of Nottingham, Nottingham NG7 2RD United Kingdom; orcid.org/0000-0001-8724-6051

[b]Marco Garavaglia - School of Life Sciences, Biodiscovery Institute, University of Nottingham, Nottingham NG7 2RD, United Kingdom

[c]Rob Van Houdt - Microbiology Unit, Belgian Nuclear Research Centre (SCK CEN), Boeretang 200, 2400 Mol, Belgium

[d]Phil Hill - School of Biosciences, The University of Nottingham, Sutton Bonington Campus, Leicestershire, LE12 5RD, United Kingdom

[a]Frankie J. Rawson - Bioelectronics Laboratory, School of Pharmacy, University of 334 Nottingham, Nottingham NG7 2RD, United Kingdom; orcid.org/0000-0002-4872-8928

[e]Katalin Kovacs - Division of Molecular Therapeutics, School of Pharmacy, University of Nottingham, Nottingham NG7 2RD, United Kingdom; orcid.org/0000-0002-0622-940X

* Email: Katalin.Kovacs@nottingham.ac.uk

Plasmid construction

To compare expression levels of the theophylline-dependent riboswitches, the P_{BAD}_Riboswitch library was constructed as follows: pMTL71301 was digested with *Sbf*I and *Xho*I; the P_{BAD} promoter and *mRFP1* were amplified via PCR from pMTL71301_P_{BAD}_*mRPF1* [44]; *mRFP1* amplification was performed with primer pairs designed with homology arms to the digested backbone (reverse) and the P_{BAD} promoter. Forward primers were also provided with a spacer sequence for insertion of the riboswitch of interest upstream of *mRFP1*. Amplification of the P_{BAD} promoter was performed with primer pairs AraC_pbad_gt_Fw/Rv. Amplification of *mRFP1* was performed using the reverse primer Rb_RFP_Rv in combination with forward primers Rb_Dx_RFP_Fw (x stands for the riboswitch of interest). All fragments were eventually assembled, thereby generating plasmids pMTL71301_P_{BAD}_RBE_*mRFP1*, pMTL71301_P_{BAD}_RBG_*mRFP1*,

pMTL71301_P_{BAD}_RBI_*mRFP1*, pMTL71301_P_{BAD}_RBI_*mRFP1*, pMTL71301_P_{BAD}_RBL_*mRFP1*, pMTL71301_P_{BAD}_RBD_*mRFP1*, pMTL71301_P_{BAD}_RBF_*mRFP1*, pMTL71301_P_{BAD}_RBH_*mRFP1*, pMTL71301_P_{BAD}_RBG_*mRFP1*, pMTL71301_P_{BAD}_RBJ_*mRFP1*.

To find a suitable promoter for expression of the sgRNA, a constitutive promoter library was built as follows. pMTL71301 was digested with *Not*I and *Nhe*I; depending on the length of the promoters used, the promoters were either amplified via PCR from a DNA template (promoter >100bp) or included as spacers in the primers for *mRFP1* amplification (promoter <100bp); amplification of *mRFP1* from pMTL71301_P_{BAD}_*mRPF1* was performed with primer pairs with the reverse primer presenting a homology arm for the digested plasmid backbone and forward primer with homology arm for the promoter under scrutiny. Isolation of promoter and/or *mRFP1* for assembly in pMTL71301 was performed as follows: P_{Pan}[41] was amplified from pUT57 template with primer pairs Pan_71301NotI_Fw/Pan_mRFP1_Rv and *mRFP1* with mRFP_Pan_Fw/mRFP1_71301NheI_Rv. λ_{Pr} was added to *mRFP1* as spacer with primer pairs λmRFP1NotI_Fw/mRFP1_71301NheI_Rv. P_{OA0284} was amplified from gDNA of *C. metallidurans* NA4 with primer pairs POA0284_mRFP1NotI_Fw/POA0284_mRFP1_Rv and *mRFP1* with mRFP_POA0284_Fw/mRFP1_71301NheI_Rv. P_{ARAE} was amplified from pRECas1- IIE[23] with primer pairs AraE_71301NotI_Fw/AraE_mRFP1_Rv and *mRFP1* with primer pairs mRFP1_AraE_Fw/ mRFP1_71301NheI_Rv respectively. J23119 was added to *mRFP1* as spacer with primer pairs mRFP1J23119NotI_Fw/ mRFP1_71301NheI_Rv and lastly, P_{trpsyn} was added to *mRFP1* as spacer with primer pairs mRFP1_Ptrpsyn_71301NotI_Fw/mRFP1_71301NheI_Rv. Eventually, plasmids pMTL71301_P_{pan}_*mRFP1*, pMTL71301_P_{λ}_*mRFP1*, pMTL71301_P_{OA0284}_*mRFP1*, pMTL71301_P_{AraE}_*mRFP1*, pMTL71301_P_{J23119}_*mRFP1* and pMTL71301_P_{trpsyn}_*mRFP1* were assembled.

For the validation experiments of the CRISPR-Cas9 system, plasmid pMTL74311 was digested with *Eco*RI and *Asc*I. *cas9* and P_{BAD}_RBI were isolated via PCR from plasmid pMTL74311_RBIC9_Δ*phaC1* (in-house plasmid for generation of knockout strains of *C. necator* H16) with primer pairs Cas9RBI_74311EcoR1_Fw/Cas9RBIJ23119_Rv. The reverse primer was designed to include the nucleotide sequence of the P_{J23119} promoter for expression of the sgRNA. the SIBS RNA scaffold was amplified with primers SIBS_J23119_Fw/ SIBS_LHAPyrE_Rv, with the forward primer having the sgRNA targeting *pyrE* and inserted as a spacer together with a unique *Spe*I restriction site. Amplification of 800bp

right and left homology arms from the genome of *C. metallidurans* CH34 was performed with primer pairs LHAPyrE_SIBS_fw/LHAPyrE_RHA_Rv. All fragments were assembled for generation of pMTL74311_RBIC9_D*pyrE*.

To build all the following plasmids for validation of the CRISPR-Cas9 system, pMTL74311_RBIC9_Δ*pyrE* was used as starting construct. pMTL74311_RBIC9_Δ*pyrE* was digested with *Asc*I/*Spe*I and gel purification of the fragments containing the CRISPR-*cas* module, and the SIBS RNA scaffold was performed as described in Materials and Methods.

The SIBS RNA scaffold of each plasmid was isolated by PCR, using the digested fragment containing the SIBS scaffold of pMTL74311_RBIC9_Δ*pyrE* as template. Primer pairs sgRNA1_RBIC9SpeI_Fw/SIBS_LHAPilA_Rv, sgRNAPilA_RBIC9_Fw/SIBS_LHAPilA_Rv, SIBSPilE_RBISpeI_Fw/SIBS_LHAPilA_Rv were then used for generation of the sgRNA targeting the *pilAE pilE* and *pilA* genes. Left and right homology arms for the plasmids targeting *pilAE, pilE* and *pilA* genes were amplified from gDNA of *C. metallidurans* CH34 using primer pairs LHAPilA_SIBS_Fw/LHAPilA_RHA_Rv & RHAPilE_LHA_Fw/RHAPilE_RBIC9AscI_Rv, LHAPilE_SIBS_Fw/LHAPilE_RHA_Rv & RHAPilE_LHA_Fw/RHAPilE_RBIC9AscI_Rv, LHAPilA_SIBS_Fw/LHAPilA_RHA_Rv & RHAPilA_LHA_Fw/RHAPilA_RBIC9_Rv, respectively. The fragments were assembled in the backbone digested with *Asc*I/*Spe*I containing the CRISPR-*cas9* module under control of the P_{BAD}_RBI construct for generation of pMTL74311_RBIC9_Δ*PilAE,* pMTL74311_RBIC9_Δ*PilE* and pMTL74311_RBIC9_Δ*PilA*. *GFP* was amplified by PCR from pUT*GFP* with primer pairs GFP_RBIC9_Fw/Rv & PrGFP_RBIC9_Fw/GFP_RBIC9_Rv for generation of *GFP* and *GFP* under control of the λ_{Pr} promoter. These were inserted between LHA and RHA of *pilA* and *pilE* genes for generation of plasmids pMTL74311_RBIC9_Δ*pilAE::GFP and* pMTL74311_RBIC9_Δ*pilAE::*λ_{Pr}*GFP.*

The nucleotide sequence of all plasmids have been deposited in the public version of the JBEI registry (https://public-registry.jbei.org) with the accession numbers reported in table S8.

```
GS_PilA      MANYPHTPTQAAKRRKETLMLQKLRNRKGFTLIELLIVVAIIGILAAIAIPQFSAYRVKA   60
CM_PilA      MTR-----QIKVVFLREQRRALERKNRRGFTLIELMIVVAIIGILAGVAIPQYQDYIARS   55
CM_PilE      -----------------------------------MIVVAIVGILAVIAVPQYQDYVARA   25
                                                :*****:**** :*:**:. * .::

GS_PilA      YNSAASSDLRNLKTALESAFADDQTYPPES------------------------------   90
CM_PilA      QFAEGMSLASGQKAGVIESFAAAGACPNNASAAVDGVPVASAIKGSYVQSVTTGGTASNA   115
CM_PilE      QFAEGLTLASAQKAAVVESFSQRGFCPSNSYSEYDGIPVSGHISGSYVQSVIVGGSATEG   85
               : . :     *:.: .:*:     * ::

GS_PilA      ------------------------------------------------------------   90
CM_PilA      -GGCTIIAKFKDEGITRGLLGKQVTLTMGNADGGSVTWQCESSADKRYVPQACAKASESG   174
CM_PilE      GVGCTITATFRTENVSPGLSGKTLTLTMFGGSSGSIAWLCKSDAENRYIPRSCT------   139


GS_PilA      --------    90
CM_PilA      KTTTTTTG    182
CM_PilE      --------    139
```

Figure S1 - Clustal Alignment of primary amino acid sequences of *pilA* from *G. sulfureducens* and *pilA/E* from *C. metallidurans* CH34.

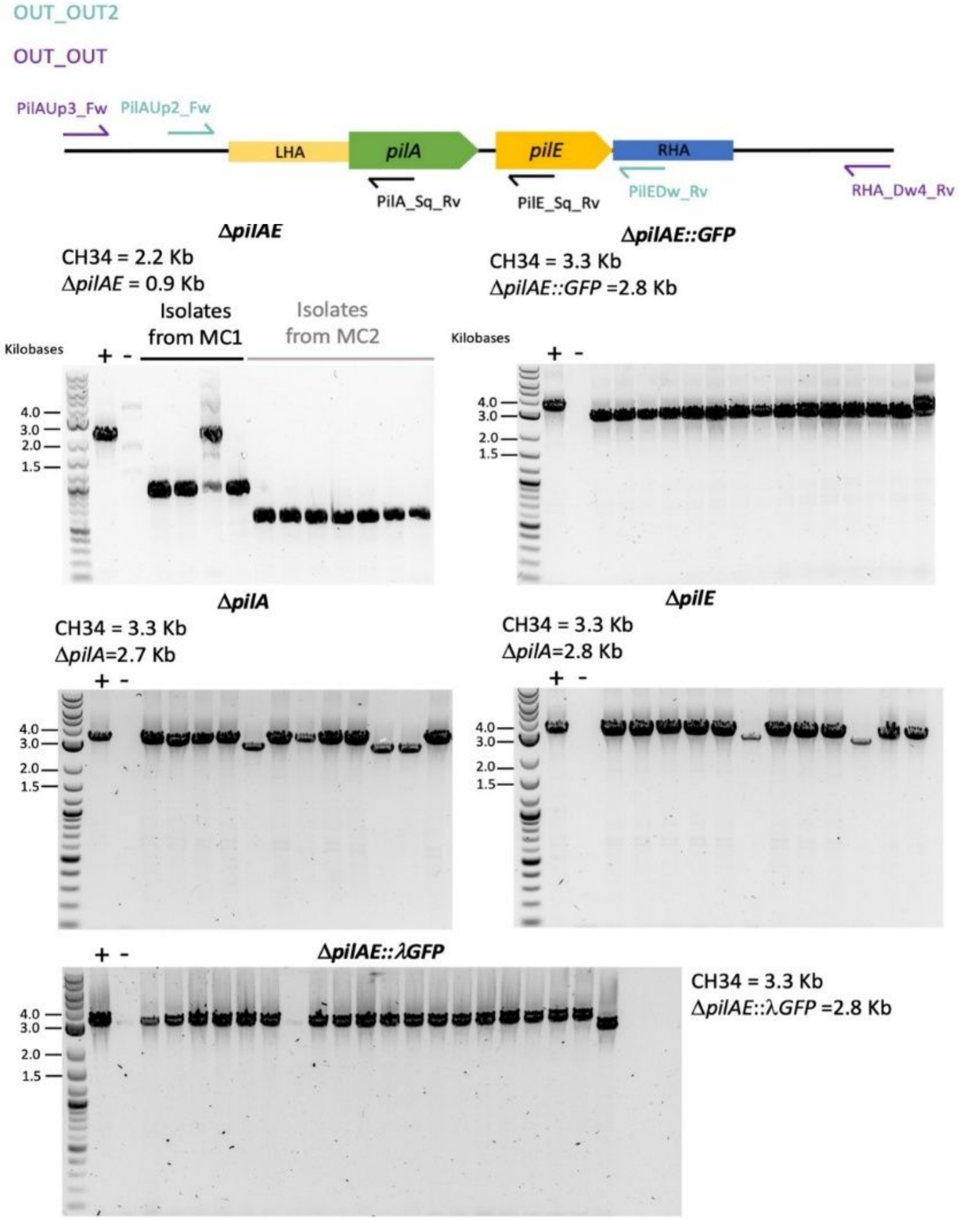

<u>**Figure S2 - cPCR strategy for identification of *C. metallidurans* CH34 Δ*pilA/E* knockout strains & agarose gels of the amplicons confirming the desired mutations**</u>. PilAUp2_Fw/PilEDw_Rv (OUT_OUT2) were used for screening of Δ*pilAE* knockouts. However, as non-specific amplification of the negative control was observed (agarose gel on the top left, lane marked with the "-" symbol), a different primer pair (PilAUp3_Fw/RHA_Dw4_Rv (OUT_OUT) was used for screening of all the other knockouts. Primers PilA_Sq_Rv and PilE_Sq_Rv were then used in combination with PilAUp3_Fw to rule out wild type contamination from Δ*pilAE,* Δ*pilA,* and Δ*pilE* strains, respectively (not shown).

Table S3 - cPCR strategy for identification of Δ*pilA/E* knockout strains of *C. metallidurans* CH34 & agarose gels of the amplicons confirming the desired mutations Table summarizing the primers combination and their screening purposes.

**Primer pairs**	**Primer name**	**Primers pair function**
**OUT_OUT**	PilA_Up3_Fw	Amplify *pilAE* locus with primers pairs binding outside homology arms
	RHA_Dw4_Rv	
**OUT_OUT2**	PilAUp2_Fw	Amplify *pilAE* locus with primers pair binding outside and inside LHA & RHA respectively
	PilEDw_Rv	
**OUT_InPilA**	PilA_Up3_Fw	Amplify *pilA* locus with primers pairs binding outside the LHA and inside *pilA*. Used to confirm pure knockout population
	PilA_sq_Rv	
**OUT_InPilE**	PilA_Up3_Fw	Amplify *pilE* locus with primers pairs binding outside the LHA and inside *pilE*. Used to confirm pure knockout population
	PilE_Sq_Rv	

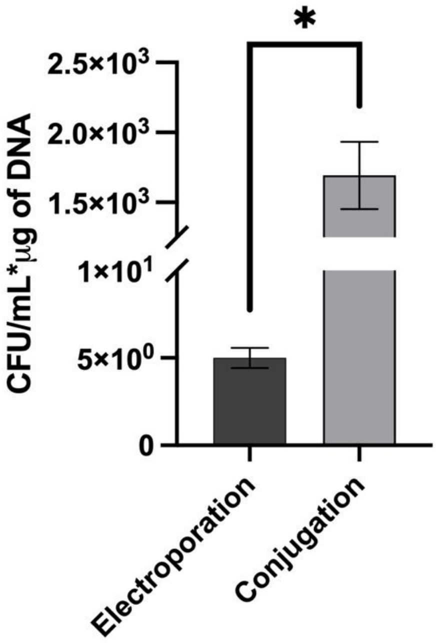

Figure S4 - Comparison between the transformation efficiencies observed following delivery of pMTL74311_P_{BAD}RBIC9_Δ*pilAE*::λ*GFP* to *C. metallidurans* CH34 by either electroporation or conjugation.

Table S5 - Illumina sequencing of *C. metallidurans* CH34 knockouts obtained with the CRISPR-Cas9 system

Strain	Type of Mutation	Region	Chromosme	Length (base pairs)	Expected deletion size (base pairs)	CDS	Gene product
CH34 Δ*pilAE*#2	Deletion	501865..503157	CHR1	1293	1293	Rmet_0472- _0473	*pilAE* type IV pili
CH34 Δ*pilAE*#3	Deletion	501865..503157	CHR1	1293	1293	Rmet_0472- _0473	*pilAE* type IV pili
CH34 Δ*pilAE*#5	Deletion	501865..503157	CHR1	1293	1293	Rmet_0472- _0473	*pilAE* type IV pili
CH34 Δ*pilA*#1	SNP (G>A)	36277	Chromid	1		Rmet_5844	Polysaccaride biosynthesis tyrosine autokinase
	SNP (G>T)	36281		1		Rmet_5844	Polysaccaride biosynthesis tyrosine autokinase
	Replacement	36277..36415	Chromid	139		Rmet_5844	Polysaccaride biosynthesis tyrosine autokinase
	SNP	501781	CHR1			Intergenic	
	Complex	802867..802931	Chromid	65		Intergenic	
CH34 Δ*pilA*#1	Deletion	501865..502413	CHR1	549	549	Rmet_0472	*pilA* type IV pili
CH34 Δ*pilA*#2	Deletion	501865..502413	CHR1	549	549	Rmet_0472	*pilA* type IV pili
CH34 Δ*pilA*#11	Deletion	501865..502413	CHR1	549	549	Rmet_0472	*pilA* type IV pili
CH34 Δ*pilE*#3	Deletion	501865..503157	CHR1	495	495	Rmet_0473	*pilE* type IV pili
CH34 Δ*pilE*#6	Deletion	501865..503157	CHR1	495	495	Rmet_0473	*pilE* type IV pili
CH34 Δ*pilE*#11	Deletion	501865..503157	CHR1	495	495	Rmet_0473	*pilE* type IV pili

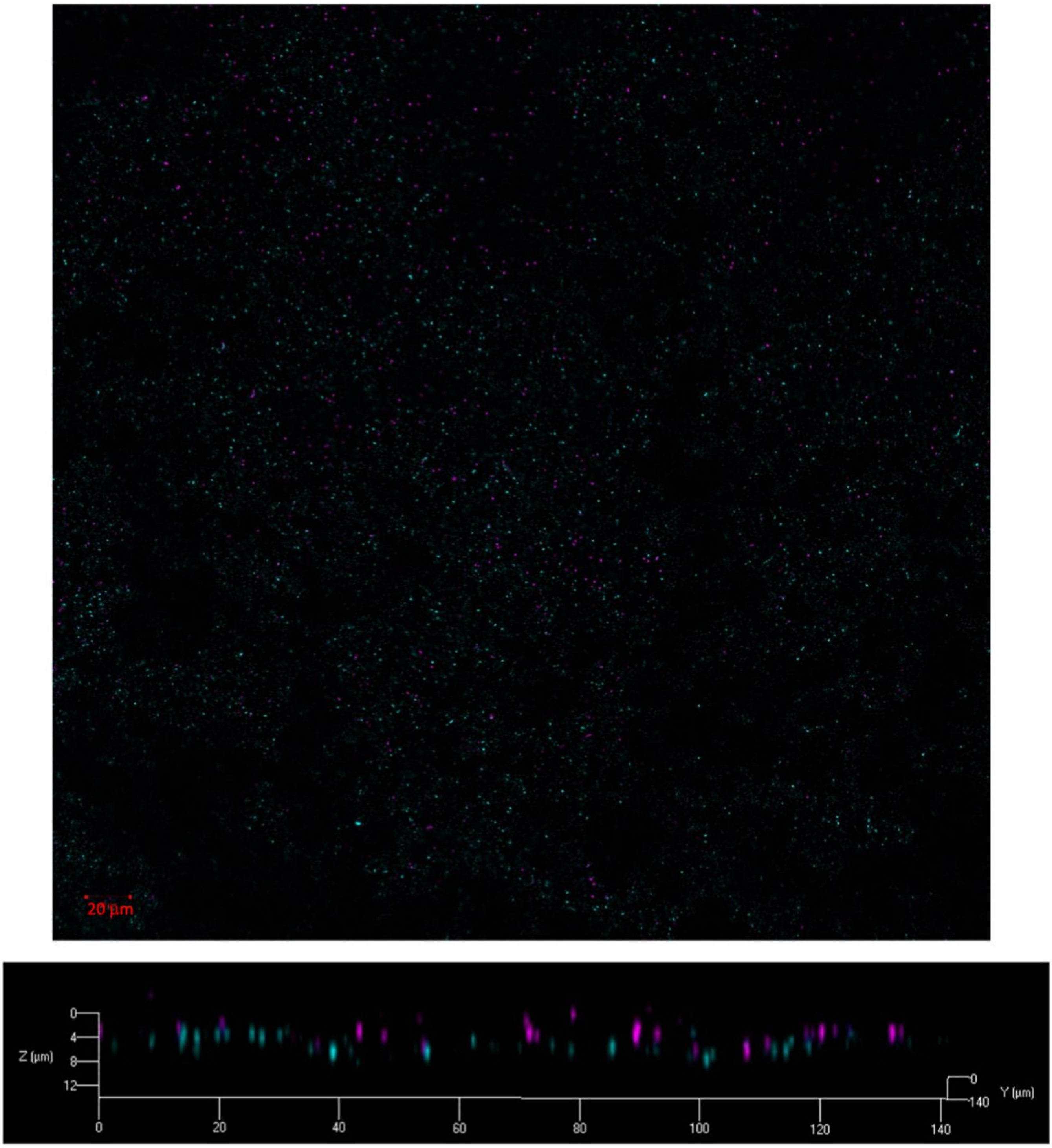

Figure S6 - Confocal microscopy of live/dead staining of the biofilm-modified SPCE with *C. metallidurans* CH34. Purple and cyan represent PI and syto9 fluorescence, respectively. The top panel represents the horizontal overview of a section of the SPCE modified with *C. metallidurans* CH34. The bottom panel shows a z-stack of the same electrode section.

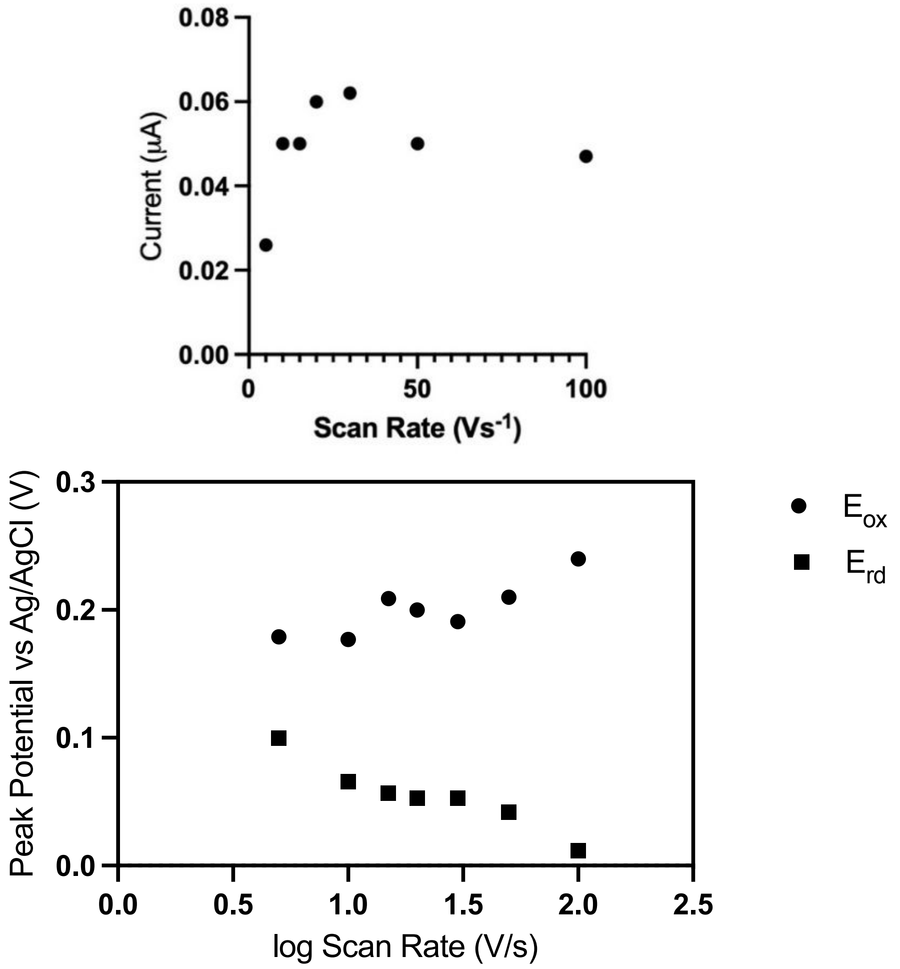

<u>Figure S7 - Scan rate experiment supporting information.</u> The top graph shows a plot of the scan rate with the anodic current peak. The bottom graph shows the plotting of the peak potentials of the anodic and cathodic currents with the logarithm of the scan rate.

Table S8 - List of plasmids and strains used in this study

	Features	Source or Reference	Accession number
Strains			
Escherichia Coli			
DH5α	Used for plasmid construction	[48]	
S17-1	Used for conjugative transfer	[49]	
Cupriavidus metallidurans		SCK-CEN	
CH34	Wild type strain	stock	
Δ*pyrE*	Derivative of CH34, Δ*pyrE*	This Study	
Δ*pilA*	Derivative of CH34, Δ*pilA*	This Study	
Δ*pilE*	Derivative of CH34, Δ*pilE*	This Study	
Δ*pilAE*	Derivative of CH34, Δ*pilAE*	This Study	
Δ*pilAE::λGFP*	Derivative of CH34, Δ*pilAE::λGFP*	This Study	
Plasmids			
pMTL71301	Tc^R, pBBR1 origin	[44]	
pMTL74311	Tc^R, IncP origin, *trfA* R271A	[44]	
pRECas1-IIRE	Cm^R*;* P_{fdx}*-E-*cas9*-P_{araE}-sgRNA-editing template (*spoIIE),* engineering of *Clostridia Sp.*	[32]	
pMTL71301_P_{BAD}RBE_*mRFP1*	Tc^R, pBBR1 origin, *trfA* R271A, P_{BAD}Riboswitch E, *mRFP1*	This study	JPUB_020557
pMTL71301_P_{BAD}RBF_*mRFP1*	Tc^R, pBBR1 origin, *trfA* R271A, P_{BAD}, Riboswitch F, *mRFP1*	This study	JPUB_020558
pMTL71301_P_{BAD}RBG_*mRFP1*	Tc^R, pBBR1 rigin, *trfA* R271A, P_{BAD}, Riboswitch G, *mRFP1*	This study	JPUB_020561
pMTL71301_P_{BAD}RBH_*mRFP1*	Tc^R, pBBR1 origin, *trfA* R271A, P_{BAD}, Riboswitch H, *mRFP1*	This study	JPUB_020562
pMTL71301_P_{BAD}RBI_*mRFP1*	Tc^R, pBBR1 origin, *trfA* R271A, P_{BAD}, Riboswitch I, *mRFP1*	This study	JPUB_020563
pMTL71301_P_{BAD}RBJ_*mRFP1*	Tc^R, pBBR1 origin, *trfA* R271A, P_{BAD}, Riboswitch J, *mRFP1*	This study	JPUB_020564
pMTL71301_P_{BAD}RBL_*mRFP1*	Tc^R, pBBR1 origin, *trfA* R271A, P_{BAD}, Riboswitch L, *mRFP1*	This study	JPUB_020565
pUC57_P_{pan}	Amp^R, pUC origin, P_{Pan} promoter	This study	
pMTL71301_*mRFP1*	Tc^R, pBBR1 origin, *trfA* R271A, *mRFP1*	This study	JPUB_020566
pMTL71301_P_{pan}_*mRFP1*	Tc^R, pBBR1 origin, *trfA* R271A, P_{Pan} *mRFP1*	This study	JPUB_020567

pMTL71301_$P_{\lambda Pr}$_*mRFP1*	Tc^R, pBBR1 origin, *trfA* R271A, $P_{\lambda Pr}$, *mRFP1*	This study	JPUB_020568
pMTL71301_P_{AraE}_*mRFP1*	Tc^R, pBBR1 origin, *trfA* R271A, P_{AraE}, *mRFP1*	This study	JPUB_020570
pMTL71301_P_{A0284}_*mRFP1*	Tc^R, pBBR1 origin, *trfA* R271A, P_{A0284}, *mRFP1*	This study	JPUB_020569
pMTL71301_P_{trpsyn}_*mRFP1*	Tc^R, pBBR1 origin, *trfA* R271A, P_{trpsyn}, *mRFP1*	This study	JPUB_020571
pMTL71301_P_{J23119}_*mRFP1*	Tc^R, pBBR1 origin, *trfA* R271A, P_{J23119}, *mRFP1*	This study	JPUB_020572
pMTL74311_P_{BAD}RBIC9_Δ*pyrE*	Tc^R, IncP origin, *trfA* R271A, P_{BAD}-E-*cas9*-J23119-sgRNA-editing template(*pyrE*), engineering *C. metallidurans* CH34, 13.7 kb	This study	JPUB_020577
pMTL74311_P_{BAD}RBIC9_Δ*pilAE*	Tc^R, IncP origin, *trfA* R271A, P_{BAD}-E-*cas9*-J23119-sgRNA-editing template (*pilAE*), engineering *C. metallidurans* CH34, 13.7 kb	This study	JPUB_020574
pMTL74311_P_{BAD}RBIC9_Δ*pilA*	Tc^R, IncP origin, *trfA* R271A, P_{BAD}-E-*cas9*-J23119-sgRNA-editing template (*pilA*), engineering *C. metallidurans* CH34, 13.7 kb	This study	JPUB_020575
pMTL74311_P_{BAD}RBIC9_Δ*pilE*	Tc^R, IncP origin, *trfA* R271A, P_{BAD}-E-*cas9*-J23119-sgRNA-editing template (*pilE*), engineering *C. metallidurans* CH34, 13.7 kb	This study	JPUB_020576

Table S8- List of plasmids and strains used in this study. Continued from previous page.

	**Features**	**Source or Reference**	**Accession number**
pMTL74311_P_{BAD}RBIC9_Δ*pilAE::GFP*	Tc^R, IncP origin, *trfA* R271A, P_{BAD}-E-*cas9*-J23119-sgRNA-editing template (*pilAE::GFP*), engineering *C. metallidurans* CH34, 13.7 kb	This study	JPUB_020578
pMTL74311_P_{BAD}RBIC9_Δ*pilAE::* λ_{Pr}*GFP*	Tc^R, IncP origin, *trfA* R271A, P_{BAD}-E-*cas9*-J23119-sgRNA-editing template (*pilAE::λGFP*), engineering *C. metallidurans* CH34, 13.7 kb	This study	JPUB_020579

Primer name	Primer sequence (5'-3')	Function	Template
Arac_pbad_gt_Fw	ccttcttcacgaggcagacctcagcctgcaggcaggataaaaaaaattgtagataaattttataaaatag	Amplification of AraC-P_{BAD} sequence including CD0164 terminator for HiFi ® assembly with Riboswitch Library. Rv primer has homology arms for universal Linker	
Arac_pbad_gt_Rv	tgagtcgtattggtaccatggagaaacagtagagag		
RbD_RFP_fw	ctactgtttctccatggtaccaatacgactcactataggttccggtgataccagcatcgtcttgatgcccttggcagcaccctgctaaggtaacaacaagatggcgagtagcgaagac	Forward amplification of RbD riboswitch for HiFi ® assembly with P_{BAD} promoter and *mRFP1*. Has homology arms for universal linker	
Rb_RFP_rv	gccagtgccaagcttgcatgtctgcaggcctcgagttaagcaccggtggagtg	General primer for reverse amplification of *mRFP1* for HiFi® assembly with pMTL710301 digested with *Xho*I	
Lk_RFP_fw	ctactgtttctccatggtaccaatacgactcactataggttccatggcgagtagcgaagac	Amplify *mRFP1* with addition of Linker (Lk)	
RbE_RFP_fw	tgtttctccatggtaccaatacgactcactataggttccggtgataccagcatcgtcttgatgcccttggcagcaccctgctaaggaggtaacaacaagatggcgagtagcgaagac	Amplification of *mRFP1* with addition of Lk_RiboswitchE	pMTL71101_P_{BAD}_*mRFP1*[44]
RbF_RFP_fw	ctactgtttctccatggtaccaatacgactcactataggttccggtgataccagcatcgtcttgatgcccttggcagcaccctgctaaggaggtaacaacatggcgagtagcgaagac	Amplification of *mRFP1* with addition of Lk_RiboswitchF	
RbG_RFP_fw	ctactgtttctccatggtaccaatacgactcactataggttccggtgataccagcatcgtcttgatgcccttggcagcaccctgctaaggaggtaacttaatggcgagtagcgaagac	Amplify *mRFP1* with addition of Lk_RiboswitchG	
RbH_RFP_fw	ctactgtttctccatggtaccaatacgactcactataggttccggtgataccagcatcgtcttgatgcccttggcagcaccctgctaaggaggtgtgttaatggcgagtagcgaagac	Amplify *mRFP1* with addition of Lk_RiboswitchH	
RbI_RFP_fw	ctactgtttctccatggtaccaatacgactcactataggttccggtgataccagcatcgtcttgatgcccttggcagcaccctgctaaggaggtcaacaagatggcgagtagcgaagac	Amplify *mRFP1* with addition of Lk_RiboswitchI	

RbJ_RFP_fw	tgtttctccatggtaccaatacgactcactataggttccggtgataccagcatcgtcttgatgcccttggcagcaccctgctaagggagtgtacgaccaatggcgagtagcgaagac	Amplify *mRFP1* with addition of Lk_RiboswitchJ

Table S9 - List of oligonucleotide primers used in this study- Linker and riboswitch sequences are highlighted in orange and blue, respectively and were added to the primer sequences as spacers.

Table S9 - List of oligonucleotide primers used in this study. Continued from previous page.

Primer name	Primer sequence (5'-3')	Function	Template
	Constitutive promoter library		
Pan_71301NotI_Fw	caggaaacagctatgaccgcgttcggtatcgaaagccg	Amplification of P_{Pan}	pUC57_P_{pan}
Pan_mRFP1_Rv	tactcgccatataacgtcctccttgtgatc		
mRFP1_Pan_Fw	aggacgttatatggcgagtagcgaagac	Amplification of *mRFP1.* Common for all constructs	
mRFP1_71301NheI_Rv	gcaggcttcttatttttatgttaagcaccggtggagtg		pMTL71101_ P_{BAD}_*mRFP1*
lmRFP1NotI_Fw	caggaaacagctatgaccgctaacaccgtgcgtgttgactattttacctctggcggtgataatggttgcatgtactaaggaggtcat	Amplification of *mRFP1* 1 promoter as spacer sequence	
PA0284_mRFP1_Fw	caggaaacagctatgaccgcgtcttcattcaaggtttttcccatggactgttgttctagtcg	Amplification of PA0284 promoter	gDNA *C. metallidurans* NA4[1]
PA0284_mRFP1_Rv	tactcgccatgcgacctcccccacgcgc		
mRFP1_PA0284_Fw	gggaggtcgcatggcgagtagcgaagac	Amplification of *mRFP1* with homology arms to PA0284	pMTL71101_ P_{BAD}_*mRFP1*
mRFP1_71301NotI_F	tactcgccatgcgacctcccccacgcgc	Cloning of promotorless *mRFP1*	pMTL71101_ P_{BAD}_*mRFP1*
AraE_71301NotI_Fw	caggaaacagctatgaccgctttatatttagtcccttgcc	Amplification of P_{AraE}	pRECas1_IIE
AraE_mRFP1_Rv	tactcgccatgaaaactcctccttaagatttatatatg		
mRFP1_AraE_Fw	aggagttttcatggcgagtagcgaagac	Amplification of *mRFP1* with homology arms to AraE	
mRFP1_J23119_71301NotI_Fw	caggaaacagctatgaccgcttgacagctagctcagtcctaggtataatgctagcaaggaggacgttatatggcgagtagcgaagac	Amplification of *mRFP1* with spacer sequence for addition of P_{J23119}	pMTL71101_ P_{BAD}_*mRFP1*
mRFP1_Ptrpsyn_71301NotI_Fw	caggaaacagctatgaccgcctgttgacaattaatcatcgaactagttaactagtacgcacaacagatcacaaggaggacgttatatggcgagtagcgaagac	Amplification of *mRFP1* with spacer sequence for addition of P_{trpsyn}	

[1] Donated by Dr. Rob Van Houdt, SCK-CEN, Mol, Belgium

Primer name	Primer sequence (5'-3')	Function	Template
	Cloning of plasmid pMTL74311_RBIC9_Δ*pyrE* & characterization of isolates		
Cas9RBI_74311EcoRI_Fw	tccatatgaccatgattacgaattctcagtcacctcctagctg	Amplification of *cas9* with P_{BAD}RBI construct with spacer for insertion of P_{J23119}	pMTL74311_Cas9_Δ*phaCI*[2]
Cas9J231119sgRNA_Rv2	gtgcaaagcggatgaaggacactagtgctagcattatacctaggactgagctagctgtcaatctagattttttttggggcg		
SIBS_J23119_ Fw	gtccttcatccgctttgcacgttttagagctagaaatagcaag	Amplification of SIBS RNA scaffold with spacer sgRNA for targeting of *pyre*	pMTL74311_Cas9_Δ*phaCI*[3]
SIBS_LHAPyrE_RV	gcttcttatttttatggcggctgaacttc		
LHAPyre_SIBS_Fw	ttttatggcggctgaacttctcgtccttgtagatc	Amplification of LHA from gDNA	gDNA *C. metallidurans* CH34 & pilin knockout
LHAPyre_RHA_Rv	gccggacgaccttagccagtttgttcaa		
RHAPyre_LHA_Fw	accttagccagtttgttcaaccagggcgcgatg	Amplify RHA from gDNA	
RHAPyre_74311AscI_Rv	atccgcgcgctggtgctgggcgcgcctcctttttgataatctca		
LHAUp_pyrE_Fw	gggatgcgcacatcacaac	cPCR of putative *pyrE* knockouts	
RHA_pyrE_Rv	gatcattgacgacgtgatctccg		
PyrE_Fw	aagatgacgcagcagacga	Primer used for Sanger sequencing of *pyrE* knockouts	
PyrE_Rv	Gatcattgacgacgtgatctccg		
	Cloning of plasmid pMTL74311_RBIC9_Δ*pilAE* & characterization of isolates		
SgRNA1_RBIC9SpeI_Fw	tcctaggtataatgctagcactagtaatgtatcgattctcggcatgttttagagctagaaatagcaagtt	Amplification of SIBS scaffold with spacers for insertion of sgRNA targeting *pilAE*	pMTL74311_RBIC9_Δ*pyrE*, *Spe*I/*Asc*I fragments
SIBS_LHAPilA_Rv	ttcatcgtcgtcgccggtcacgccataaaaataagaagcctgcatttg		
LHAPilA_SIBS_Fw	ttatttttatggcgtgaccggcgacgacgatg	Amplification of LHA to *pilAE*	gDNA *C. metallidurans* CH34
LHAPilA_RHA_Rv	aaggcgcgccgttgaccccttttcatttgtgttgtgc		
RHAPilE_LHA_Fw	aggggtcaacggcgcgccttaatccgggggcggtctag	Amplification of RHA to *pilAE*	
RHA_RBIC9AscI_Rv	tgagattatcaaaaaggaggctttccagcagcagcacc		
	pMTL74311_RBIC9_Δ*pilA* & characterization of isolates		
sgRNAPilA_RBIC9_F	ggtataatgctagcactagtcgactcactcgc	Amplification of	pMTL74311_RBIC9

[2] In-house plasmid for generation of KO strains of *C. necator* H16, not published

w	tttcgcgcagggttttagagctagaaatagcaagtta	SIBS scaffold with spacers for insertion of sgRNA targeting *pilA*	_Δ*pyrE*, *Spe*I/*Asc*I fragments
SIBSPilA_LHA_Rv	tcgccggtcacgccataaaaataagaagcctg		
LHAPilA_SIBS_Fw	//	Amplification of LHA to *pilA*	gDNA *C. metallidurans* CH34
LHAPilA_SIBS_Rv	tccatatgaccatgattacgaattctcagtcacctcctagctg		
RHAPilA_LHA_Fw	aaaggggtcaacggtagctagcattacgtcc	Amplification of RHA to *pilA*	
RHAPilA_AscI_Rv	ggtcatgagattatcaaaaaggaggcgcgccaaggtgttcttcgaaccag		

Table S9 - List of oligonucleotide primers used in this study. Continued from previous page.

Primer name	Primer sequence (5'-3')	Function	Template
pMTL74311_RBIC9_Δ*pilE* & characterization of isolates			
SIBSPilE_RBISpeI_Fw	tcctaggtataatgctagcactagtgcagtagcggctccatcgcgtgggttttagagctagaaatagcaag	Amplification of SIBS scaffold with spacers for insertion of sgRNA targeting *pilA*	pMTL74311_RBIC9_Δ*pyrE*, *Spe*I/*Asc*I fragments
SIBSPilE_LHA_Rv	cgattctttccgccataaaaataagaagcc		
LHAPilE_SIBS_Fw	ttttatggcggaaagaatcgtcgaggcttc	Amplification of LHA to *pile*	gDNA *C. metallidurans*CH34
LHA_PilE_Rv	cccggattaaatgacccccctgggaagttg		
RHAPilE_LHA_Fw	agggggtcatttaatccgggggcggtctag	Amplification of RHA to *pile*	
RHAPilE_RBIC9AscI_Rv	//		
pMTL74311_RBIC9_Δ*pilAE*::*GFP* & characterization of isolates			
GFP_RBIC9_Fw	aaatgaaaaggggtcaacggatgcgtaaaggagaagaac	Amplification of *GFP* for substitution with *pilAE*	pUT*GFP* (Donated by Rob Van Houdt, SCK-CEN, Mol, Belgium)
GFP RBIC9_RV	agaccgcccccggattaaggttatttgtatagttcatccatgc		
pMTL74311_RBIC9_Δ*pilAE*::λ*GFP* & characterization of isolates			
PrGFP_RBIC9_Fw	aaatgaaaaggggtcaacggtaacaccgtgcgtgttgactattttacctctggcggtgataatggttgcatgtactaaggaggtcatatgcgtaaaggagaagaac	Amplification of λ*GFP* for substitution with *pilAE*	pUT*GFP* (Donated by Rob Van Houdt, SCK-CEN, Mol, Belgium)
Screening of knockout strains of *C. metallidurans* CH34			
PilA_Up2_Fw	gagcctgccgtacctgaaga	Fw primer binds outside *pilA* LHA. Rv primer binds inside *pilE* RHA binds outside the homology arms for identification of knockouts	gDNA *C. metallidurans*CH34 & knockout strains
PilEDw_Rv	ccatccgctggttcgaagaa		
PilA_Up3_Fw	ccagcagaagaaggccattttctgg		
RHA_Dw4_Rv	gaaacctgatcaccggactggagtt		
PilA _sq_Rv	ttacccgactcactcgctttcg	Binds inside *pilA*. Knockout screening	
PilE_Sq_Rv	atcgaggaatgtatcgattctcgg	Binds inside *pilE*. Knockout screening	

C9_cPCR_Fw	Confirmation of loss of
C9_cPCR_Rv	the editing plasmid

Table S9 - List of oligonucleotide primers used in this study. Continued from previous page

Table S10 - List of spacer sequences included in primers used in this study

Spacer target	Spacer oligo sequence (5'-3')	Plasmid reference
pyrE	Gtccttcatccgctttgcac	pMTL74311_RBIC9_Δ*pyrE*
pilAE/pilAE::gfp/pilAE::λgfp	Aatgtatcgattctcggcat	pMTL74311_RBIC9_Δ*pilAE/*
		pMTL74311_RBIC9_Δ*pilAE::GFP/*
		pMTL74311_RBIC9_Δ*pilAE::λGFP*
pilA	Cgactcactcgctttcgcgcagg	pMTL74311_RBIC9_Δ*pilA*
PilE	Gcagtagcggctccatcgcgtgg	pMTL74311_RBIC9_Δ*pilE*
